# Exploring the Adsorption and Reactions of Methyl Radicals on M(111) Surfaces (M=Cu, Ag, Au): A DFT Study

**DOI:** 10.1002/cphc.202400979

**Published:** 2025-02-25

**Authors:** Pankaj Kumar, Dan Meyerstein, Amir Mizrahi, Haya Kornweitz

**Affiliations:** ^1^ Chemical Sciences Department The Radical Reactions Research Center Ariel University Ariel Israel; ^2^ Chemistry Department Ben-Gurion University Beer-Sheva Israel; ^3^ Nuclear Research Centre Negev Beer-Sheva Israel

**Keywords:** Methyl radicals, DFT, Coinage metals, Heterogeneous catalysis, Ethane production

## Abstract

It was reported that adsorbed methyl radicals produce ethane with Ag^0^‐ and Au^0^‐nanoparticles in aqueous media, whereas on Cu^0^‐powders, the product is methanol. The source of these differences was explored computationally, using the DFT method. The results indicate that up to six radicals can be adsorbed on Ag(111) and Au(111), (top site), while only four can be adsorbed on Cu(111) (fcc site), each surface containing eight atoms. The diffusion of the radicals on the surface is very easy on silver and copper, as this is achieved with a very low barrier (0.06 eV and 0.15 eV for Ag(111) and Cu(111), respectively), while on Au(111), the barrier is higher, 0.51 eV. The formation of ethane via a reaction of two adsorbed radicals is thermodynamically plausible for all studied coverage ratios on the three surfaces, but kinetically, it is plausible at room temperature only on Au(111) and Ag(111) at full coverage. Ethane can also be produced on Au(111) and Ag(111) by a collision of a solvated radical and an adsorbed radical. This is a barrierless process. On Cu(111), the yield of such a process is CH_4_(aq), and an adsorbed CH_2_ which reacts further with a non‐adsorbed water molecule to produce adsorbed CH_3_OH.

## Graphical abstract

Methyl radicals, in both aqueous and gaseous phases, spontaneously adsorb onto M(111) (M=Cu, Ag, Au) surfaces. The adsorbed radicals react via the Langmuir‐Hinshelwood mechanism only on gold and silver surfaces at high coverage to produce ethane. Additionally, an Eley‐Rideal mechanism is also possible, producing ethane on Ag(111) and Au(111), and methanol (CH_3_OH) on Cu(111) in an aqueous solution.

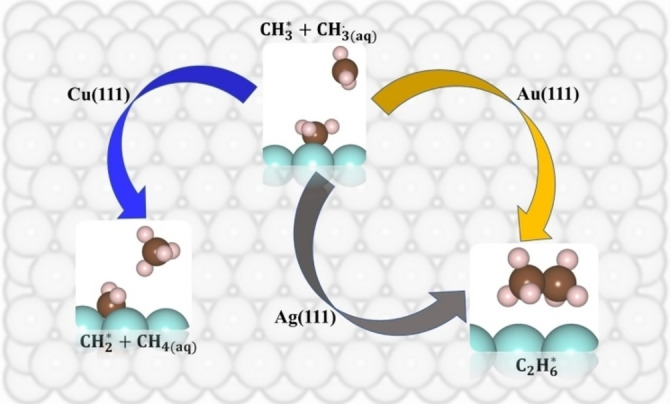

## Introduction

The study of reactions involving radicals has advanced significantly over the past four decades,[^1^, ^2^, ^3^, ^4^, ^5^, ^6^] and it is now regarded as a practical and adaptable technique. Radicals are involved in many processes catalyzed by nanoparticles (NP), such as electrochemistry,[^7^, ^8^, ^9^, ^10^, ^11^, ^12^] energy storage,^[13]^ photo‐catalysis,[^13^, ^14^, ^15^] solar cells,[^16^, ^17^] hydrogen evolution^[18]^ and catalytic de‐halogenation process.^[19]^ In organic synthesis, radicals are used for the production of fine chemicals,[^20^, ^21^, ^22^, ^23^, ^24^, ^25^, ^26^] polymers,[^27^, ^28^] medicines,^[29]^ and natural products.[^30^, ^31^] Alkyl radicals play a crucial role in many important processes, including catalysis,[^32^, ^33^] photo‐chemistry,^[34]^ advanced‐oxidation‐technologies,[^35^, ^36^, ^37^] and many more. Methyl radicals, being the most elementary alkyl radicals, are commonly employed in mechanistic investigations.^[38]^ Methyl radicals are involved in the production of methanol and formaldehyde[^39^, ^40^] from methane, and higher hydrocarbons^[41]^ in the gas phase.^[42]^ The formation of carbon‐carbon bonds is enhanced by the presence of alkyl radicals, which are effective intermediates in the process.^[43]^ Noble‐metal and metal oxide nanoparticles (M^0^−NPs, M=Ag,^[44]^ Au,^[44]^ TiO_2_,[^45^, ^46^] and Cu^0[47]^) catalyze the rapid reaction in which adsorbed methyl radicals produce ethane through surface‐catalyzed dimerization.[^44^, ^45^, ^46^, ^48^] Reactions of alkyl radicals are very important in biology. They are often produced by a rapid reaction with hydroxyl radicals.^[38]^ The interaction of alkyl radicals with organic substrates plays a crucial role in numerous enzymatic reactions.[^49^, ^50^, ^51^, ^52^] Methyl radicals are involved in oxidative stress processes and play a role in the onset of various health issues.[^52^, ^53^, ^54^] Previous studies have demonstrated that the simple methyl radical is adsorbed at the surface M^0^‐NPs via a covalent bond,[^46^, ^55^] then, a catalytic dimerization occurs, resulting in the production of ethane.[^44^, ^45^, ^47^, ^48^] This process is described in equations (1)–(3);1, 2, 3

(1) $$M^{0}-NPs+{CH}_{3}\to {(M}^{0}-NPs)-{CH}_{3}$$

(2) $${(M}^{0}-NPs)-{\left({CH}_{3}\right)}_{n-1}+{CH}_{3}\to {(M}^{0}-NPs)-{\left({CH}_{3}\right)}_{n}$$

(3) $${(M}^{0}-NPs)-{\left({CH}_{3}\right)}_{n}\to {(M}^{0}-NPs)-{\left({CH}_{3}\right)}_{n-2}+C_{2}H_{6}$$

The rapid reaction of methyl radicals with nanoparticles is greatly influenced by the reaction medium and other co‐adsorbates.[^44^, ^45^, ^56^] This process was studied experimentally on Au, and Ag, with ethane as the final product^[57]^ but still, the different catalytic behaviour of these different metallic NPs is not clear; therefore, it was decided to study the mechanism of this process computationally using DFT. Surprisingly when Cu^0^(powders) replace Cu^0^‐NPs ethane is not formed and the final product is methanol.^[58]^ Little is known about the mechanism of reaction (3) that can, in principle, be replaced by reaction 4.

(4) $${(M}^{0}-NPs)-{\left({CH}_{3}\right)}_{n}+{CH}_{3}\to {(M}^{0}-NPs)-{\left({CH}_{3}\right)}_{n-1}+C_{2}H_{6}$$

The experimental results favored reaction (3) but could not rule out reaction (4).

In this study, the plausibility of the formation of ethane is explored on the noble‐coinage metals Cu, Ag, and Au. Adsorption energies, charge transfer, and activation energy barriers are considered to elucidate the mechanism of ethane formation. The ethane production through both Langmuir‐Hinshelwood (LH) – reaction of two adsorbed radicals (reaction 3), and Eley‐Rideal (ER) mechanisms ‐ reaction of an adsorbed radical with a solvated one (reaction 4), was explored. The research focused on the aqueous media, but a parallel study was performed also in gaseous media for comparison.

## Computational Methods

The Vienna ab initio Simulation Package (VASP)[^59^, ^60^] was used to conduct first principle calculation within the framework of density functional theory (DFT).^[61]^ To deal with electron‐ion core focused interfaces, the projector augmented wave (PAW) has been used.^[62]^ An energy cut‐off of 500 eV was employed for each slab. A Monkhorst‐Pack mesh of 6×6×1 k‐point was used to sample the Brillouin zone.^[63]^ These values were verified to give the required accuracy with 10^−3^ eV‐Å^−1^ and 10^−5^ eV convergence criteria for forces and energy. These criteria were used for all the calculations, including optimization and transition state calculations. The Perdew‐Burke‐Ernzerhof (PBE)[^64^, ^65^] pseudopotential was used to manage electron exchange and correlation. The DFT‐D2 dispersion correction by Grimme[^66^, ^67^] was used to describe the long‐range van der Waals (vdW) interactions. The calculations were done without pressure (PSTRESS =0). Non‐spin‐polarized calculations were performed. The solvent effect was considered using an implicit self‐consistent electrolyte solvation model, VASPsol^[68]^ using the default parameters as implemented in VASP, these parameters are benchmarked for solvation in water, with the default value of 78.4 for the relative permittivity. Thus, the accurate ground‐state structures were obtained. The reaction transition states (TS) were located using the Climbing Image Nudged Elastic Band method (Cl‐NEB)^[69]^ by considering five images between the initial state (IS) and the final state (FS)

All M(111) surfaces were modeled using a six‐layer slab; each layer consists of eight metal atoms, and a vacuum region of 16 Å was introduced between the slabs in the z‐direction to avoid unwanted interactions. In the calculations in the aqueous phase, the vacuum region is replaced with aqueous media using VASPsol. The harmonic oscillator approximation was used to perform phonon calculations for each optimized structure, using 0.015 Å step width to obtain the zero‐point vibration energies (ZPVE) of the system. These calculations were used to validate that the optimized structures are real minima or TS (one additional imaginary frequency). The ZPVE values were also used to calculate Gibbs free energy. The VESTA code[^70^, ^71^, ^72^] was used to illustrate the stable structures and the TS. The adsorption energy (*E_ads_*) of adsorbate on the M(111) surfaces was calculated using equation 5:
(5)

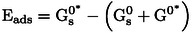



(6)

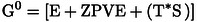




E is the electronic energy, T represents the absolute room temperature (298.15 K), and S represents the entropy. $G_{s}^{0^{*}}$ is the free energy of the surface with the adsorbate, $G^{0^{*}}$ is the free energy of the M(111) surface, and $G_{s}^{0}$ is the free energy of the isolated adsorbate. Negative $E_{ads}$ values mean that the adsorption is favored, and vice‐versa. The free energy (${\Delta G}^{o}$ ) of a reaction was calculated using the equation:

(7) $$\;{\Delta G}^{0}={\sum G}_{products}^{0}-{\sum G}_{reactants}^{0}\;$$

Calculating the quantity of charge transfer was done using the Bader charge analysis.[^73^, ^74^] Positive CT values mean CT from the surface to the adsorbate, while negative values mean CT from the adsorbate to the surface. We determined the rate constants (k) through the application of the Arrhenius equation,^[75]^

(8) $$\;k={Ae}^{-E_{a}/k_{B}T}$$

Where $E_{a}$ is the activation energy barrier, $k_{B}$ is the Boltzmann constant, *A* is the pre‐exponential factor, and T is the absolute room temperature. In this context, we have utilized the pre‐exponential factor (*A*) value of 10^10^ (the rate constant of diffusion‐controlled reactions in aqueous solutions) to determine the rate constants.

## Results and Discussion

### I Adsorption of Methyl Radicals

#### Methyl Radical Adsorption On M(111) Surfaces

**Figure 1:**
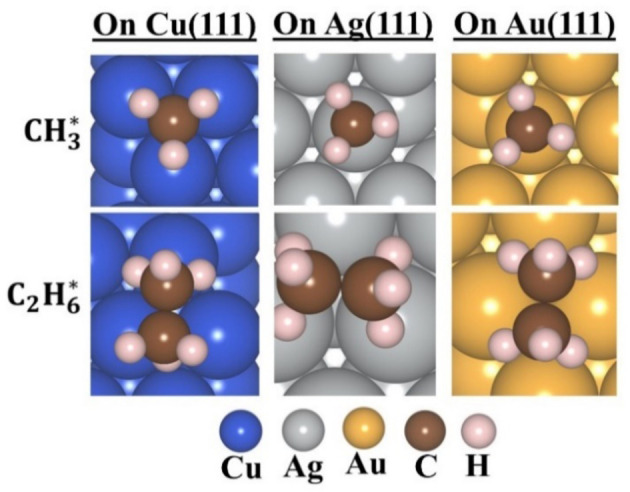
Optimized geometries, at the best adsorption site, of adsorbed methyl radical and ethane on the different M(111) surfaces.

The largest number of methyl radicals that can be adsorbed on M(111) surfaces containing eight metal atoms in each layer, was found to be six. In a low coverage ratio (1/6), the best adsorption site for the methyl radical is the atop position on Au(111) and Ag(111) and a fcc site on Cu(111), while ethane is adsorbed at a bridge site on Au(111) and at a hollow site on Ag(111) and Cu(111). The structures of the adsorbed CH_3_ and C_2_H_6_ at their best adsorption sites are given in Figure 1, and the adsorption energies, charge transfer, and binding distances are tabulated in Table 1 for the aqueous phase; the adsorption energies values for the gaseous medium are given in Table S1. The optimized structures of methyl radicals and ethane on M(111) surfaces at all adsorption sites are depicted in Tables S4, S5, S6, and S7.

**Table 1:** Adsorption energies (E_ads_), charge transfer (e), and the shortest distance between M(111) and C atoms in the aqueous phase, at room temperature.

Metals	Adsorbate	Adsorption site	Adsorption energy $\left(eV\right)$	Charge transfer^[a]^ (e)	M(111)‐C distance (Å)
Cu	${CH}_{3}$	fcc	−2.28	0.31	2.19
	$C_{2}H_{6}$	hcp	−0.45	0.03	3.10
Ag	${CH}_{3}$	top	−1.87	0.23	2.17
	$C_{2}H_{6}$	hcp	−0.23	0.03	3.14
Au	${CH}_{3}$	top	−2.32	0.05	2.12
	$C_{2}H_{6}$	bridge	−0.81	0.01	3.41

[a] Positive values indicate charge transfer from the surface to the methyl radical and ethane.

In Table 1 adsorption energies, charge transfer, and the shortest M−C (M=Ag, Au, Cu) distances for methyl radicals and ethane at their best adsorption sites are given. The adsorption of the methyl radicals is significantly stronger (−1.87 eV–−2.32 eV) than the adsorption of ethane (−0.23 eV–−0.81 eV). The weak adsorption of ethane is reflected in the long M−C bonds (3.10 Å–3.41 Å) for adsorbed ethane, in comparison to the M−C distance for adsorbed methyl radicals (2.12 Å–2.19 Å). The adsorption of both methyl radicals and ethane is the strongest on Au(111), then on Ag(111), and the weakest on Cu(111). The adsorption energies are −1.87 eV and −0.23 eV, and −2.32 eV and −0.81 eV for methyl radicals and ethane on Ag(111) and Au(111), respectively, differ slightly from the previously calculated values,^[57]^ −1.81 eV and −0.40 eV, and −2.44 eV and −0.73 eV, for these species on the same metals, respectively. These differences are attributed to small changes in the adsorption sites; the methyl radicals and the ethane are bulky species, and their adsorption is very sensitive to their exact position on the surface as depicted in Tables S8 and S9. Lower values for the adsorption energy of methyl radicals were published previously on Cu(111) −1.28 eV,^[76]^ on Ag(111) −0.92 eV^[77]^ and on Au(111) −1.19 eV.^[78]^ These significant differences can be attributed to different computational details and considerations of dispersion correction and ZPVE. Similar values for the CT to the methyl radical and ethane on Ag(111) and Au(111) surfaces were found in ref.,^[57]^ 0.28 e for CH_3_/Ag(111), 0.05 e for C_2_H_6_/Ag(111) (0.23 e and 0.03 e in this study), 0.08 e for CH_3_/Au(111), and 0.01 e for C_2_H_6_/Au(111) (0.05 e and 0.01 e in this study).^[57]^ It is known^[57]^ that the adsorption energy decreases as the CT increases, this is the case for silver and copper, the adsorption energy of CH_3_ on Cu(111) (−2.28 eV) is lower than on Ag(111) (−1.87 eV), and the CT on Cu(111) (0.31 e) is larger than on Ag(111) (0.23 e), but the CT from Au(111) to the adsorbate is very small, 0.05 e for CH_3_ and 0.01 e for C_2_H_6_, and both adsorbates are best adsorbed on Au(111) (lowest adsorption energy). Tables S2 and S3 display the charge transfer values for methyl radicals and ethane at all adsorption sites in both media.

#### Methyl Radical's Adsorption at Higher Coverage

**Figure 2:**
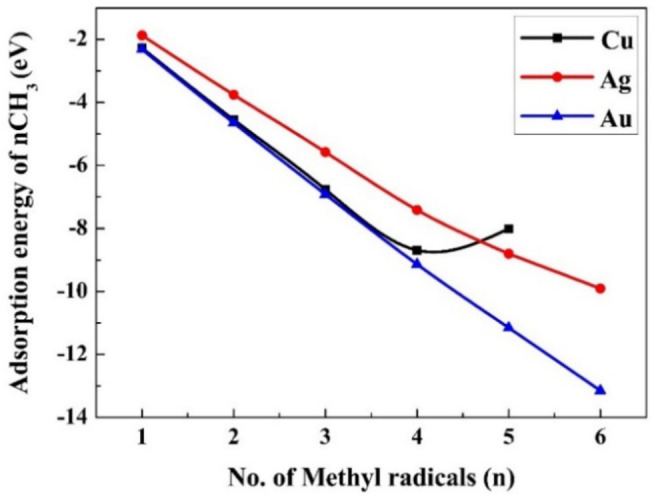
The adsorption energy (eV) of n (n=1–6) methyl radicals on M(111) surfaces.

In this section, the adsorption energies of methyl radicals as a function of the coverage ratio of methyl radical adsorbed on the M(111) surfaces are evaluated. The total adsorption energy for n (n=1–6 for silver and gold, and n=1–5 for copper) methyl radicals for the three M(111) surfaces is depicted in Figure 2 (Figure S1 for gaseous phase) and Table S18 (Table S19 for gaseous phase). The total adsorption energies decrease (become more negative) monotonically on Ag(111) and on Au(111), but on Cu(111), the value increases for the fifth methyl radical indicating that the adsorption of the fifth radical is not favored. The adsorption of an additional methyl radical on a surface with (n‐1) (n=1–6) pre‐adsorbed methyl radicals, forming n adsorbed radicals, according to reaction 9, was studied to evaluate the thermodynamic limit of the coverage ratio.
(9)

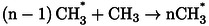




**Figure 3:**
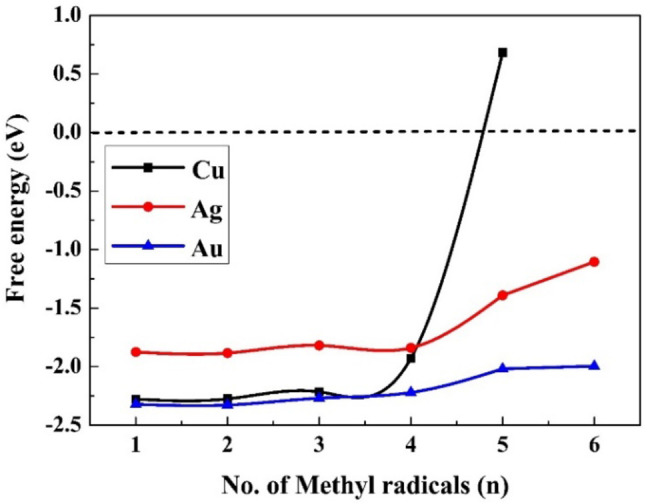
The free energies (${\Delta G}^{0}$ ) of reaction 9 in the aqueous phase on M(111) surfaces.

The number of adsorbed methyl radicals was increased step by step by adding one adsorbed methyl radical at the nearest fcc or atop site, respectively. The addition of adsorbed methyl radicals continued until the free energy of adsorption (${\Delta G}^{0}$ (9)), for reaction (9) became endergonic. The values of ${\Delta G}^{0}$ are given in Figure 3, and the results in the gas phase are given in Figure S2. In aqueous media up to six methyl radicals can be adsorbed on the surface and only five in the gaseous phase; adsorption of another methyl distorts the whole structure, as shown in Tables S10 and S11. In low coverage, the lowest adsorption energies are obtained for gold and the highest for silver, but for gold and silver the values of ${\Delta G}^{0}$ of reaction 9 are only slightly increasing with the coverage ratio, while for copper, the value increases significantly for n=5, and becomes positive, meaning that thermodynamically, the adsorption of five methyl radicals on Cu(111) is not plausible; no more than four methyl radicals can be adsorbed on the surface. The optimized structure n=(1–6 for Ag and Au, and 1–4 for Cu) methyl radicals on M(111) surfaces are given in Tables S10 and S11. The adsorption energies of *n* methyl radicals on M(111) surfaces in the gas phase are depicted in Figure S1. The charge transfer for all the adsorbed methyl radicals is presented in Figures S7 and S8, while the charge transfer on each methyl radical (CT) that has been adsorbed is shown in Figures S9 and S10 in the aqueous and gaseous phases, respectively. The shortest distance between M(111) and carbon (C) atoms under different surface coverage ratios is given in Figures S5 and S6 for the aqueous and gaseous phases.

### II Langmuir‐Hinshelwood Mechanism

#### Production Of Ethane via a Reaction of Two Adsorbed Methyl Radicals

The dimerization of two methyl radicals, according to reaction 10, in their best adsorption sites to form ethane, was considered on all M(111) surfaces.
(10)

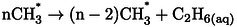




**Figure 4:**
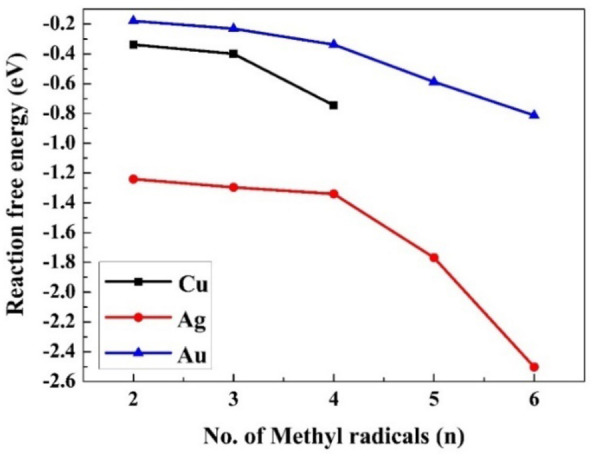
The reaction free energies (${\Delta G}^{0}$ ) for the evolution of ethane on the M(111) surface in the aqueous phase according to reaction (10).

Here *n* is the number of methyl radicals present on the M(111) surface. The reaction free energies of the ethane evolution as a function of n are given in Figure 4 for the aqueous media and in Figure S3 for the gaseous phase. The values of the free energy of reaction 10 on the various M(111) surfaces are depicted in Table 2 (Table S20 for the gaseous phase). The activation energies of reaction 10 at different n values are presented in Table 3. These barriers are too high to produce ethane in low coverage of ${CH}_{3}$ at room temperature.

**Table 2:** The evaluated reaction free energies (ΔG^0^) values of the number of methyl radicals on M(111) surfaces for the ethane evolution in the aqueous phase.

Number of ${CH}_{3}$ adsorbed on M(111) surface	${\Delta G}_{C_{2}H_{6}\left(aq\right)}^{0}$		
	Cu(111)	Ag(111)	Au(111)
2	−0.34	−1.24	−0.18
3	−0.40	−1.30	−0.23
4	−0.75	−1.34	−0.34
5	–	−1.77	−0.59
6	–	−2.50	−0.81

**Table 3:** The E_a_ barriers and rate constants (k) for the C_2_H_6_ evolution on M(111) surfaces in aqueous solution.

Metals	No. of ${CH}_{3}$ on M(111)	E_a_ (eV)	Rate constants (M^−1^s^−1^)
Cu	2	1.76	1.61×10^−20^
	4	1.56	4.35×10^−17^
Ag	2	1.52	2.15×10^−16^
	5	0.94	1.41×10^−06^
	6	0.69	2.01×10^−02^
Au	2	1.01	7.00×10^−08^
	5	0.59	1.25×10^0^
	6	0.43	6.18×10^2^

According to the results in Figure 4, the largest exergonicity for this process was observed for Ag(111) and then Cu(111), the values for Au(111) are significantly lower. The exergonicity becomes larger (more negative) as the coverage ratio increases. The reaction free energies ΔG^0^ for ethane production decrease from 1 to n (n=6 for Au and Ag, and 4 for Cu), for Cu(111) from −0.34 to −0.75 eV, for Ag(111) from −1.24 to −2.50 eV, and for Au(111) from −0.18 to −0.81 eV.

**Figure 5:**
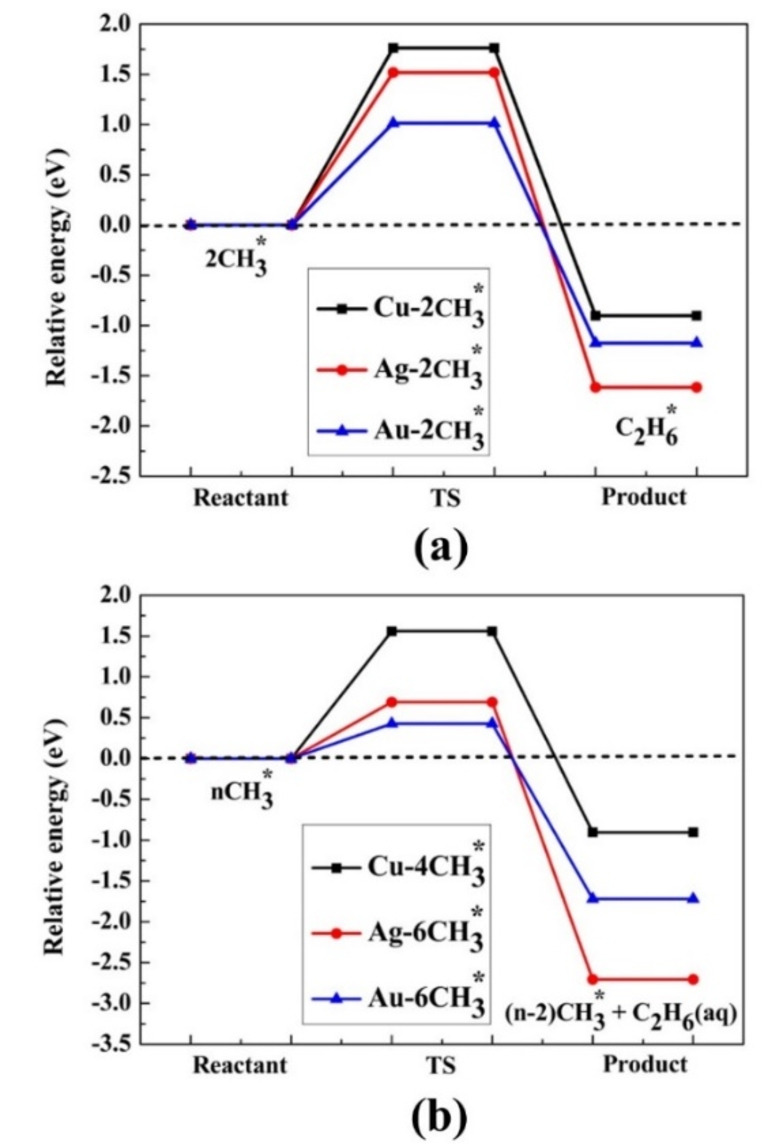
The E_a_ of (a) 2${CH}_{3}$ and (b) 4&6${CH}_{3}$ are placed on the M(111) surface for the evolution of ethane in the aqueous phase.

Although all the values in Figure 4 are exergonic, and the production of ethane is plausible thermodynamically, the kinetics of this reaction were studied to evaluate whether ethane is practically produced. According to the high activation energies and low‐rate constants given in Table 3 and depicted in Figure 5a; ethane is not produced in a low coverage ratio (2/6). As the exergonicity of reaction 10 increases with the coverage ratio (the values are lower), the activation barriers are expected to decrease; therefore, activation energy barriers were also computed at full coverage (full coverage is 6/6 for gold and silver and 4/5 for copper). The results are given in Table 3 and depicted in Figure 5b. The results in the gaseous phase are given in Table S21 and shown in Figure S4. The activation energy barrier decreases when the coverage ratio increases; for Cu(111) in an aqueous solution, the decrease is from 1.76 to 1.56 eV, for Ag(111), from 1.52 to 0.69 eV, and for Au(111), from 1.01 to 0.43 eV. These values indicate that the production of ethane is practically possible only on the Au(111) surface, as the barrier for full coverage is moderate (0.43 eV) and the rate constant is higher, but it may occur also on Ag(111) in the case of full coverage. Indeed, experimental results^44^ indicate the evolution of ethane on Au‐NPs and Ag‐NPs. To note, the barriers in the gaseous phase (Table S21) are lower than for the aqueous phase (Table 3), except for Au(111), where they are very high in the gaseous phase. The lowest barriers in the gaseous phase were found on Ag(111), 0.75 eV, and 0.72 eV for 2/6 and 5/6 coverage ratios, respectively. These are moderate values that enable a slow release of ethane, in the gaseous phase at room temperature.

**Figure 6:**
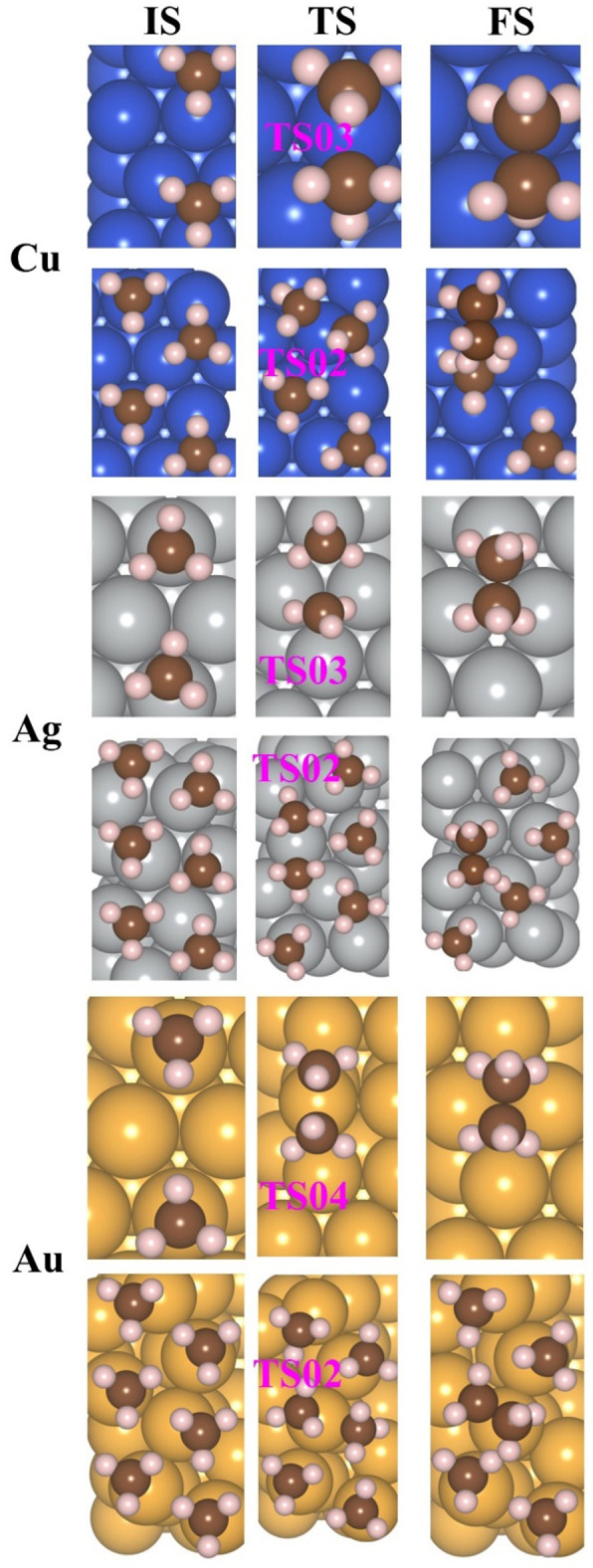
All structures of initial state (IS), final state (FS), and TS of 2, 4 or 6${CH}_{3}$ for the evolution of ethane.

All the structures of initial state (IS), final state (FS), and transition state (TS) are given in Figure 6 for Cu(111), Ag(111), and Au(111), respectively. The structures for the gaseous phase are given in Tables S12, S13, and S14, respectively.

#### The Diffusion of Adsorbed Methyl Radicals on M(111) Surfaces

**Figure 7:**
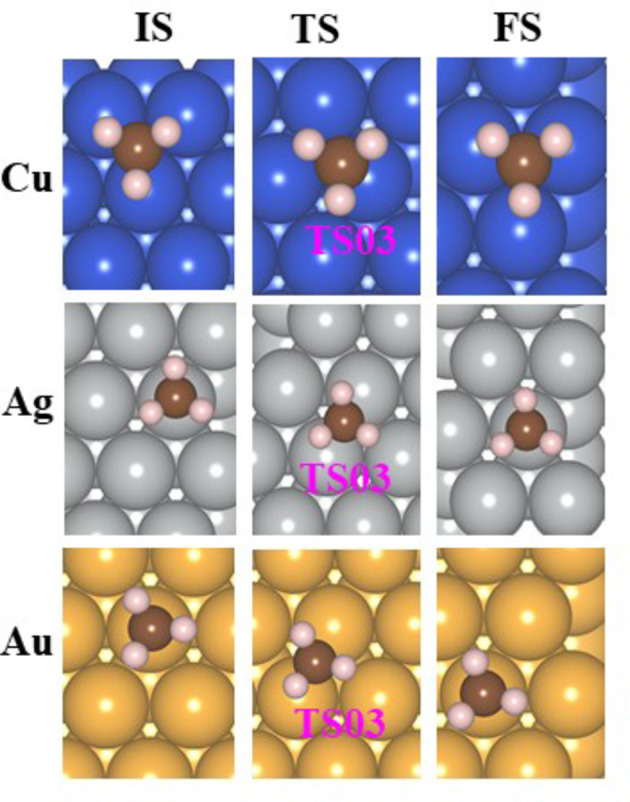
The structures of initial state (IS), final state (FS), and TS of diffusion of ${CH}_{3}$ on the M(111) surface for aqueous phase.

The energy barrier to produce ethane depends on the precise location of the adsorbed methyl radicals on the surface. As it is expected that the methyl radicals are adsorbed randomly at their best adsorption sites on the M(111) surfaces, they should move on the surface till they reach the desired configuration for the reaction. Therefore, the barrier for the diffusion of an adsorbed methyl radical from one site to another identical site was calculated. The diffusion barriers and rate constants for aqueous medium are given in Table 4. The diffusion barriers for the gaseous phase are given in Table S22. All the structures of the initial state (IS), final state (FS), and TS are given in Figure 7 for Cu(111), Ag(111), and Au(111), respectively.

**Table 4:** The diffusion barriers and rate constants for the movement of ${CH}_{3}$ adsorbed on M(111) surfaces from initial to final position in aqueous medium.

Metals	E_a_ (eV)	Rate constants (M^−1^s^−1^)
Cu	0.15	2.82×10^7^
Ag	0.06	1.10×10^9^
Au	0.51	2.52×10^1^

The lowest barrier was computed for Ag(111) (0.06 eV), in line with its highest adsorption energy (−1.87 eV). The adsorption energies of ${CH}_{3}$ on gold and copper are similar (−2.32 eV and −2.28 eV, respectively) but the diffusion barrier on Au(111) is higher, 0.51 eV, in comparison to 0.15 eV for Cu(111). The difference in their diffusion barriers can be attributed to the different best adsorption sites of the methyl radicals on both surfaces (atop site for Au and fcc for Cu). The diffusion barrier of methyl radicals that were studied previously: on Cu(111) 0.05 eV^[76]^ and on Ag(111) 0.11 eV^[77]^ are in good agreement with our values. We have seen that ethane evolution is plausible on gold; the calculated barrier for this reaction at full coverage is 0.43 eV (Table 3); as the diffusion barrier on gold is 0.51 eV, it is the rate‐determining step in the production of ethane. The diffusion barrier on silver is low, therefore, it will not affect the production of ethane. Although the diffusion barrier on Cu(111) is low (0.15 eV), the barrier for the reaction of two methyls on the Cu(111) surface is high; therefore, ethane is not produced via reaction 10.

### III Eley‐Rideal Mechanism

#### Ethane Production via One Adsorbed Methyl Radical and Another One in Solution

The results in the previous section indicate that ethane can be produced on M(111) surfaces only on Au(111) and Ag(111) at high coverage. However, experimental results point out that these M^0^‐NPs catalyze the formation of ethane.[^44^, ^45^] In this section the production of ethane is evaluated in another scenario; a methyl radical in the aqueous solution is approaching an adsorbed methyl and producing ethane according to reaction 11. It is of interest to note that an analogous mechanism was reported for the reaction of methyl radicals with L_n_Co^III^‐CH_3_.^[79]^

(11)

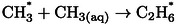




Surprisingly, in this case, ethane is formed without an energy barrier on Ag^0^ and Au^0^; methyl radicals move randomly in the solution; if, during their movement, a radical hits an adsorbed methyl, the production of ethane occurs, as the rate constant for diffusion‐controlled reaction in aqueous solution is 10^10^, this is a fast process. This means that the assumption that *k_11_* is negligible when calculating the rate constants of the reaction of ${CH}_{3}^{.}$ radicals with Ag^0^‐NPs & Au^0^‐NPs^44^ is wrong, and the production of ethane on Ag^0^‐NPs & Au^0^‐NPs^44^ is mainly via reaction 11.

**Figure 8:**
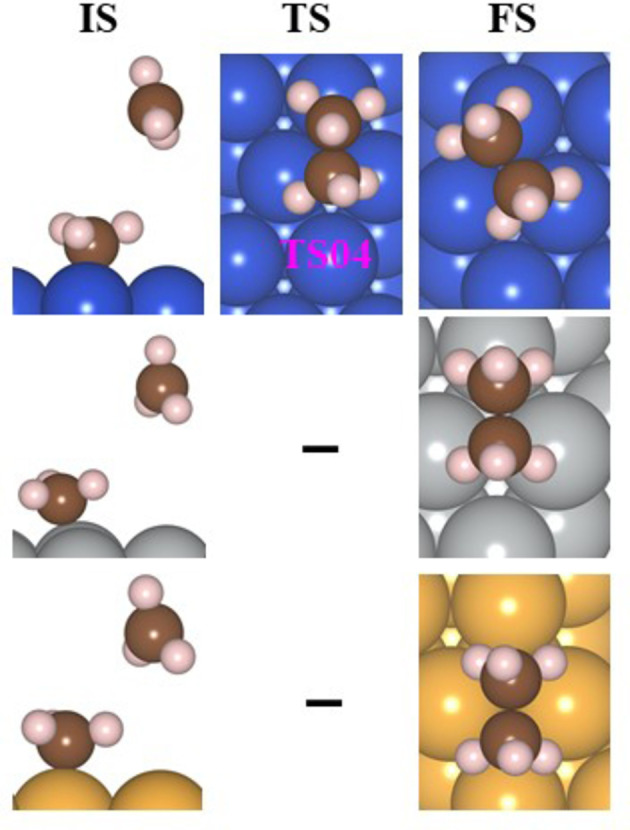
The structures of the initial state (IS), final state (FS), and TS of reaction 11 on the M(111) surface for the evolution of ethane in the aqueous phase.

The reaction of an adsorbed methyl and methyl in solution on Cu(111) is highly exergonic (ΔG^0^=−2.98 eV). On the Cu(111) surface a two‐step process occurs: first, CH_4_ (methane) is formed in the solution, above the surface, without a barrier, and 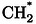 is formed on the surface, in a highly exergonic (ΔG^0^=−0.81 eV) reaction. This means that the adsorption of the methyls on copper affects dramatically its properties as methyl radicals abstract hydrogen atoms from saturated alkanes slowly.^[6]^ Then ethane is formed with a barrier of 0.63 eV (rate constant=2.80×10^−1^ M^−1^s^−1^). This scenario in which CH_2_* and CH_4_(aq) are formed on the Cu(111) surface is given in Figure S11 (Table S15). The figures of the IS, FS, and TS for the formation of ethane on Cu(111) are depicted in Figure 8. According to the barrier, the rate constant for the formation of ethane is lower than the diffusion in aqueous solution by nine orders of magnitudes (2.80×10^−1^ M^−1^s^−1^ in comparison to 10^10^ M^−1^s^−1^) therefore, the methane is expected to move away prior to the formation of ethane and this process is not plausible.

#### Reaction with Water and Formation of Ch_3_oh on Cu(111) Surface

12

(12)

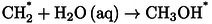




**Figure 9:**
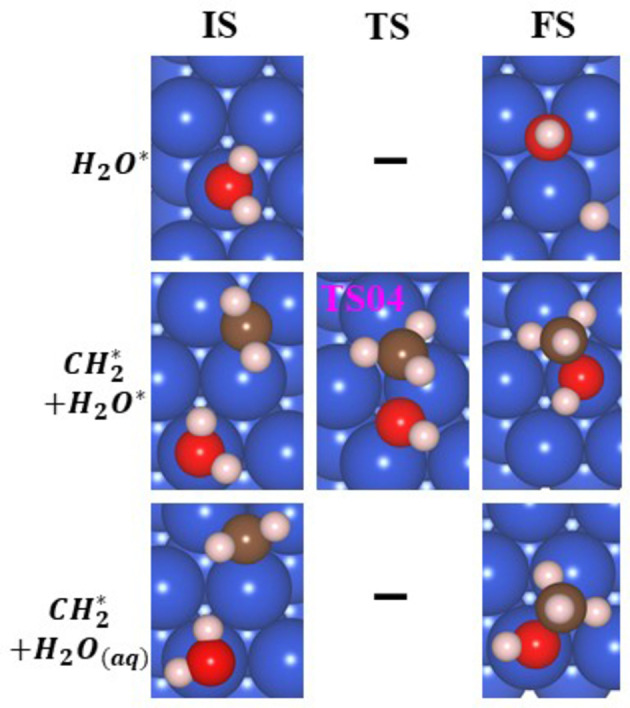
The structures of the initial state (IS), final state (FS), and TS of the optimized geometries (top view) for the splitting of water, the reaction of CH_2_* with H_2_O* to produce methanol (CH_3_OH), and the reaction of 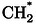 with H_2_O(aq) to produce CH_3_OH.

The 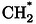 that is formed on the Cu(111) can react with water to give methanol. The splitting of water into OH+H on the Cu surface does not occur due to its endergonic nature (ΔG^0^=0.03 eV); therefore, the reaction of 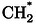 with 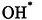 or 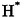 is not considered. The IS and FS of the splitting of water are given in Figure 9.

The reaction of Cu with adsorbed water and 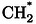 is exergonic (ΔG^0^=−0.38 eV). This is a two‐step process; in the first step, the reactants are rotated with a barrier of 0.11 eV, and then CH_3_OH is formed with a high barrier of 1.35 eV. Due to the large barrier, this reaction is not expected to occur. Methanol can be formed by a reaction with H_2_O from the solution. This is a barrierless exergonic reaction (ΔG^0^=−0.67 eV). The reactant, product, and TS of the reaction of 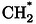 with 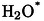 and H_2_O(aq) are depicted in Figure 9. The production of methanol, as found on powdered copper^[58]^ is via the reaction 12 with H_2_O(aq). The energy path for the reaction of 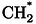 with 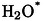 on the Cu(111) surface is given in Figure S12. This is the mechanism of methanol production on powdered copper.^[58]^

## Conclusions

The adsorption of methyl radicals on M(111) surfaces was explored, revealing that up to six methyl radicals can be adsorbed on Ag(111) and Au(111) surfaces; they are best adsorbed at the top site. On Cu(111), only four radicals can be adsorbed; their best adsorption site is fcc. The diffusion of the radicals on the surface is almost barrierless for silver (0.06 eV), the barrier is very low for copper (0.15 eV), and higher for gold (0.51 eV). The ${CH}_{3}$ radicals can move on the surface and reach the best configuration to react and produce ethane. The production of ethane according to the LH mechanism is exergonic for all coverage ratios, but the barriers are relatively high; therefore, kinetically, in the aqueous phase, ethane can be produced at room temperature only on gold and silver at a high coverage ratio; the barrier, in this case, is 0.69 eV for silver and 0.43 eV for gold. A slow production of ethane is expected on the Ag(111) surface in the gaseous phase at room temperatures, as the barriers for this process are 0.72 eV–0.75 eV in different coverage ratios. Ethane can be produced in the ER mechanism; one ${CH}_{3}$ radical that is moving randomly in the aqueous solution hits an adsorbed CH_3_ radical on the surface. This process is barrierless on all surfaces, therefore, the ER is the preferred mechanism. In this scenario, ethane is produced on gold and silver. On copper, the methyl radical in the solution abstracts hydrogen from the adsorbed methyl and forms CH_4_. The adsorbed 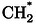 thus formed reacts further in a barrierless reaction, with a water molecule from the solution to produce methanol. This is the mechanism of formation of methanol reported experimentally.^[58]^ However, on Cu^0^‐NPs ethane formation was reported.^[48]^ The source of the latter observation might be that the nanoparticles were polycrystalline.

## Conflict of Interests

The authors declare no conflict of interest.

1

## Supporting information

As a service to our authors and readers, this journal provides supporting information supplied by the authors. Such materials are peer reviewed and may be re‐organized for online delivery, but are not copy‐edited or typeset. Technical support issues arising from supporting information (other than missing files) should be addressed to the authors.

Supporting Information

## Data Availability

The data that support the findings of this study are available in the supplementary material of this article.
