# Supplementary material for: Exploring the Adsorption and Reactions of Methyl Radicals on M(111) Surfaces (M=Cu, Ag, Au): A DFT Study
Source: Chemphyschem. 2025 Feb 25;26(8):e202400979. doi: 10.1002/cphc.202400979 (PMC12005131; doi:10.1002/cphc.202400979)
Supplement: Supplementary file 1 — Supporting Information [file CPHC-26-e202400979-s001.pdf]

ChemPhysChem

Supporting Information

Exploring the Adsorption and Reactions of Methyl Radicals on M(111) Surfaces (M=Cu, Ag, Au): A DFT Study

Pankaj Kumar, Dan Meyerstein, Amir Mizrahi, and Haya Kornweitz*

Supplementary Data

Exploring the Adsorption and Reactions of Methyl Radicals on M(111) Surfaces (M = Cu, Ag, Au): A DFT Study

Pankaj Kumar[a], Dan Meyerstein[a, b], Amir Mizrahi[c] and Haya Kornweitz*[a]

[a] Chemical Sciences Department, The Radical Reactions Research Center, Ariel University, Ariel, Israel
E-mail: hayak@ariel.ac.il

[b] Chemistry Department, Ben-Gurion University, Beer-Sheva, Israel

[c] Nuclear Research Centre Negev, Beer-Sheva, Israel

Figures

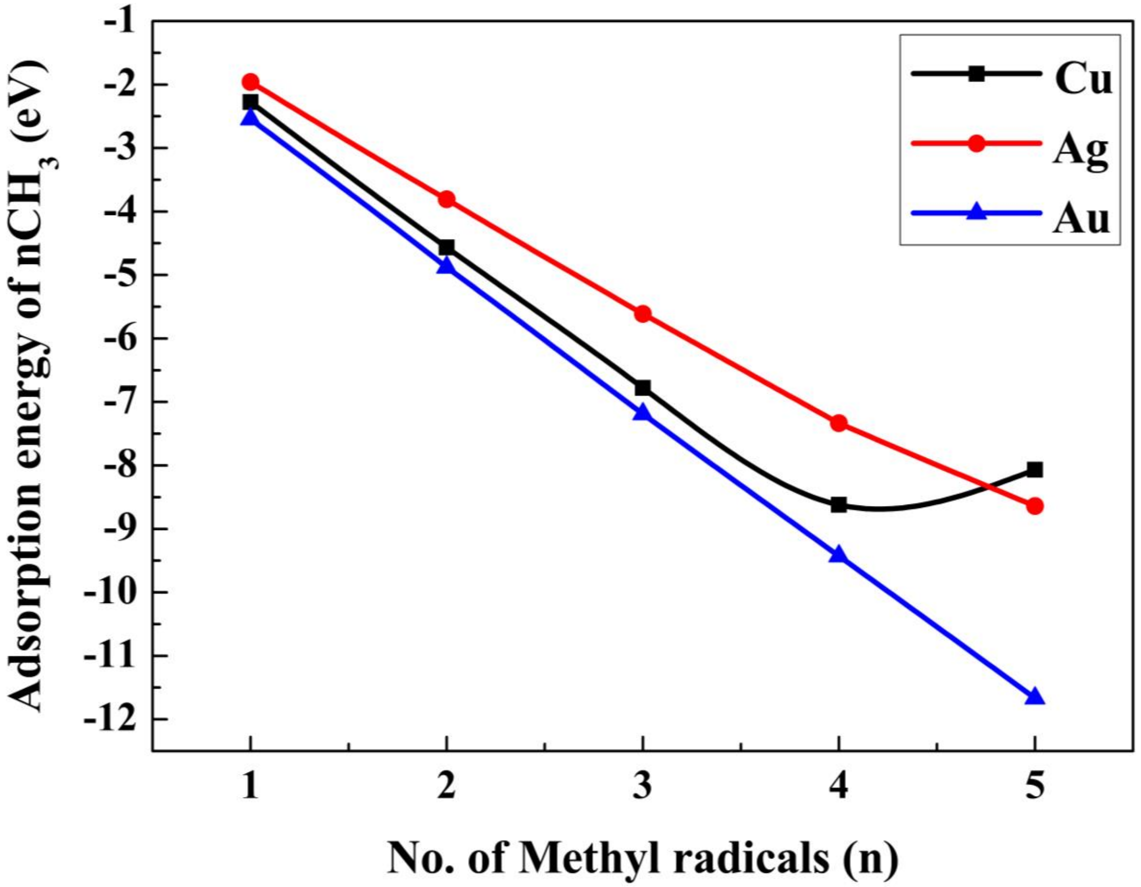

Figure S1: The adsorption energies (E_{ads}) of n methyl radical on various M(111) surfaces in the gaseous phase.

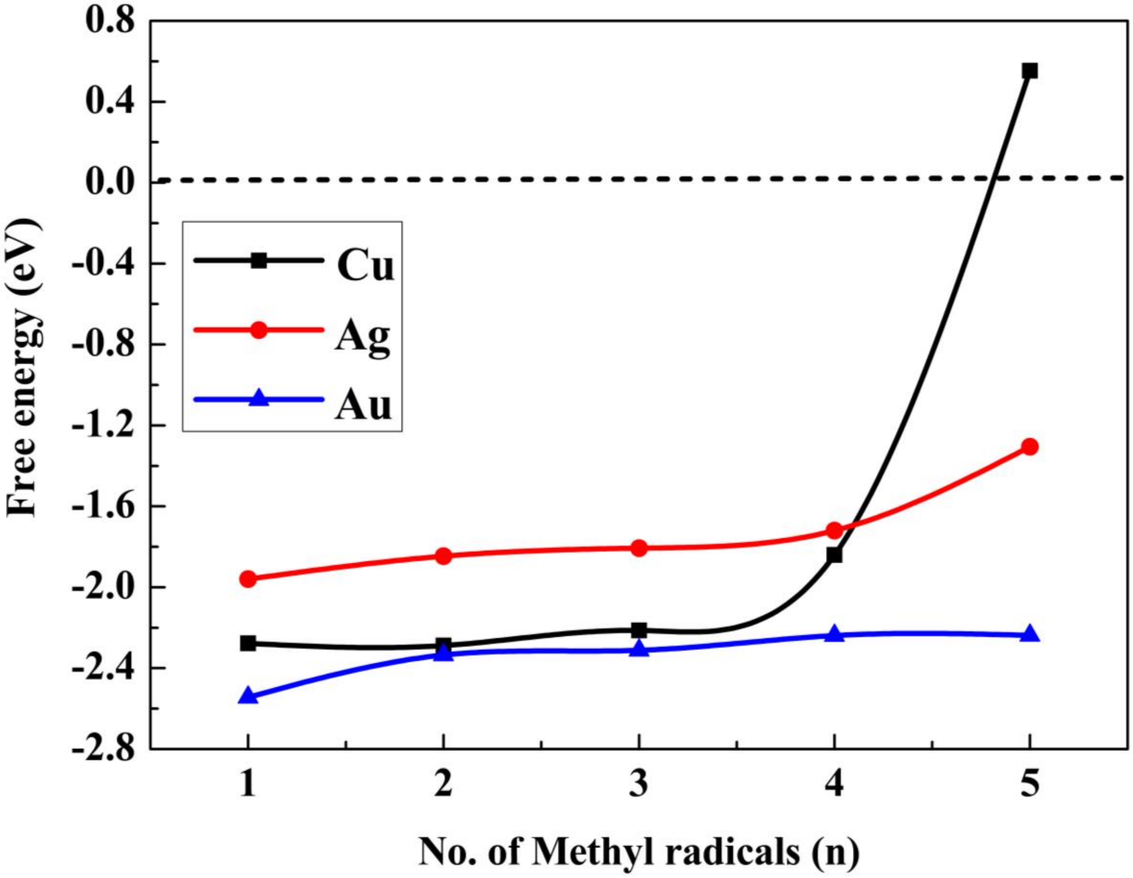

Figure S2: The free energies ($\Delta G^0_{CH_3(g)}$) of n methyl radicals on various M(111) surfaces in the gaseous phase (using **equation (9)**).

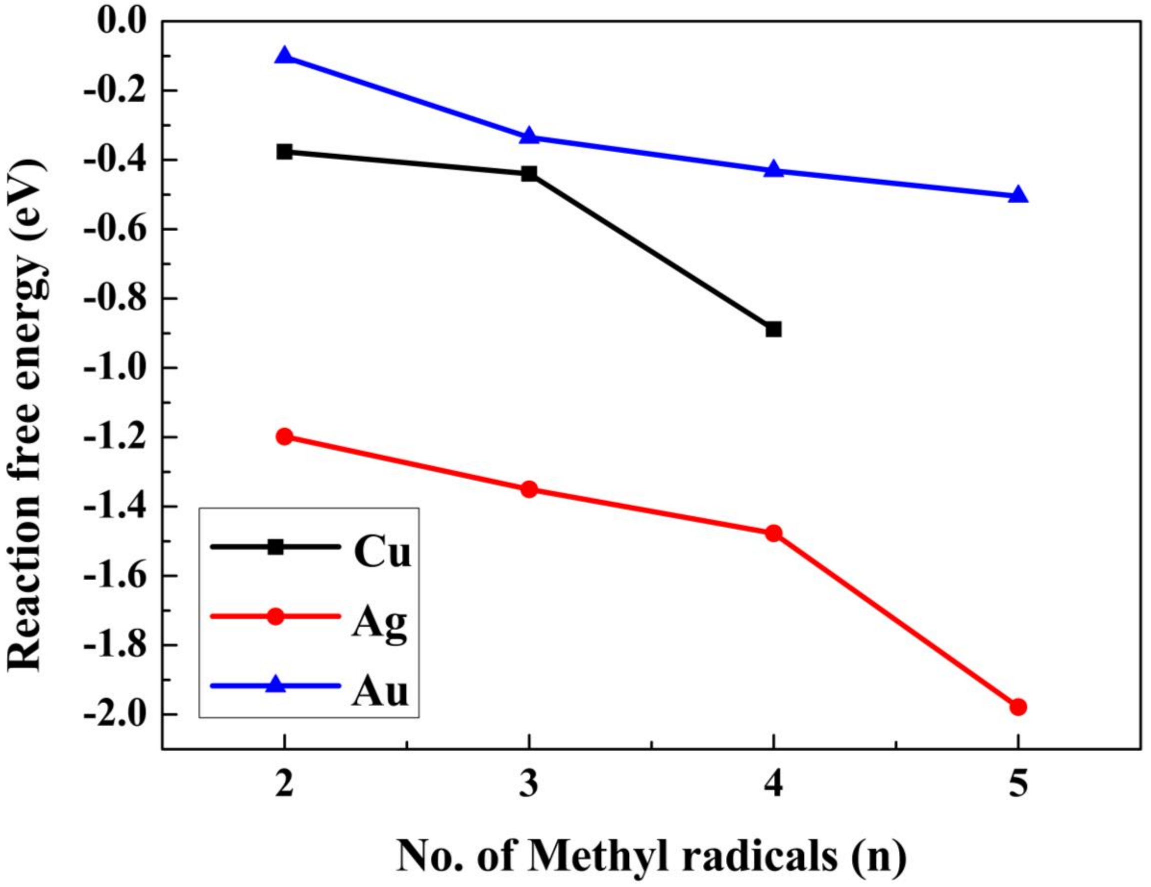

Figure S3: The reaction-free energies ($\Delta G^0_{C_2H_6(g)}$) for the evolution of ethane on the M(111) surfaces in gaseous phase (using **equation (10)**).

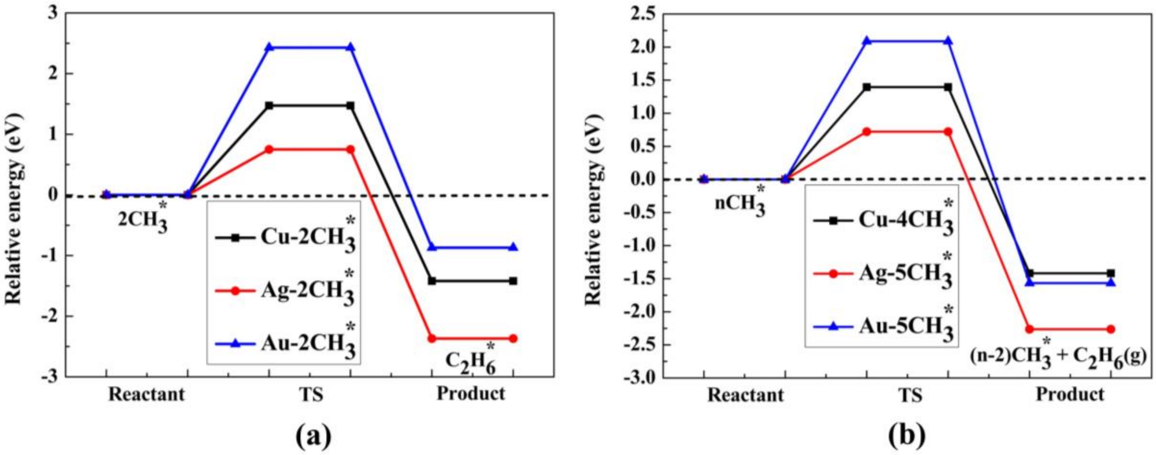

Figure S4: The E$_a$ of (a) $2CH_3$ and (b) 4 & $5CH_3$ on the M(111) surfaces for the evolution of ethane in the gaseous phase.

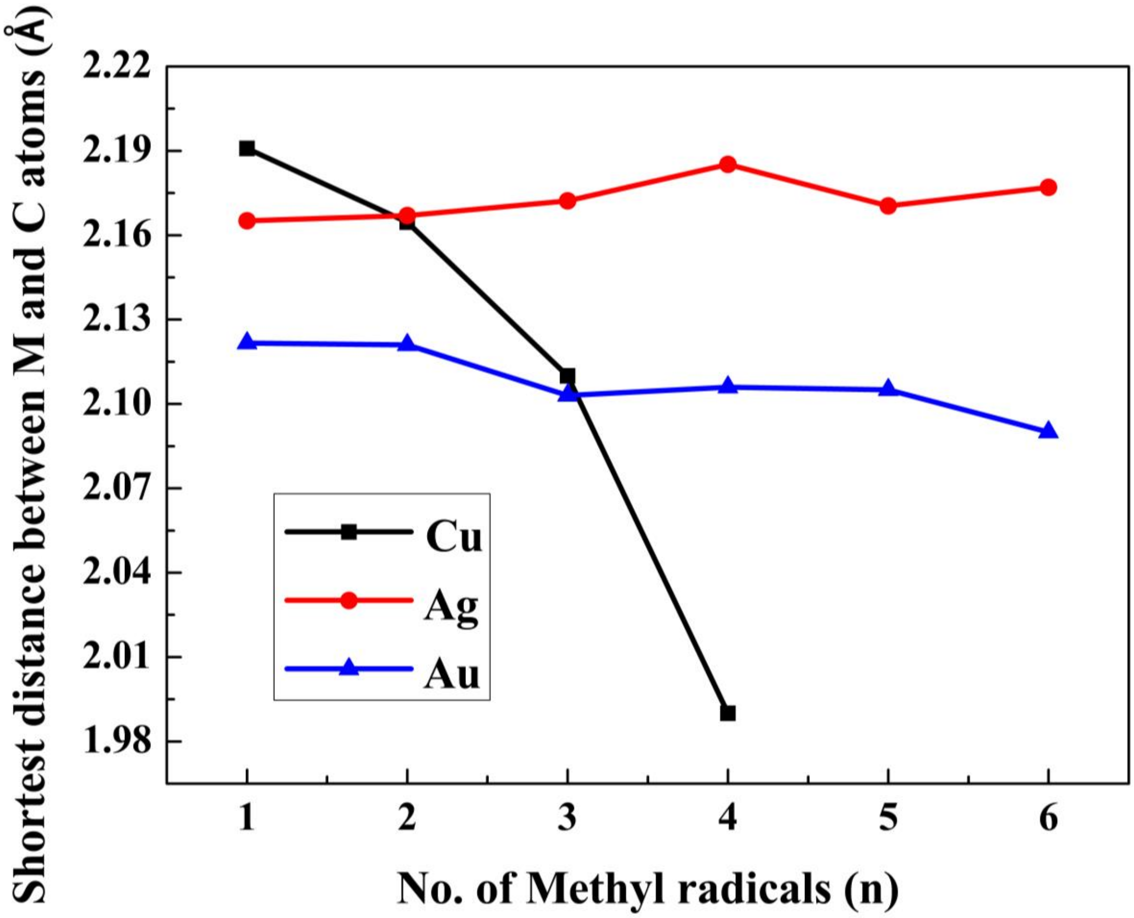

Figure S5: The shortest distance between M and carbon atoms (Å) under different surface coverage in the aqueous phase.

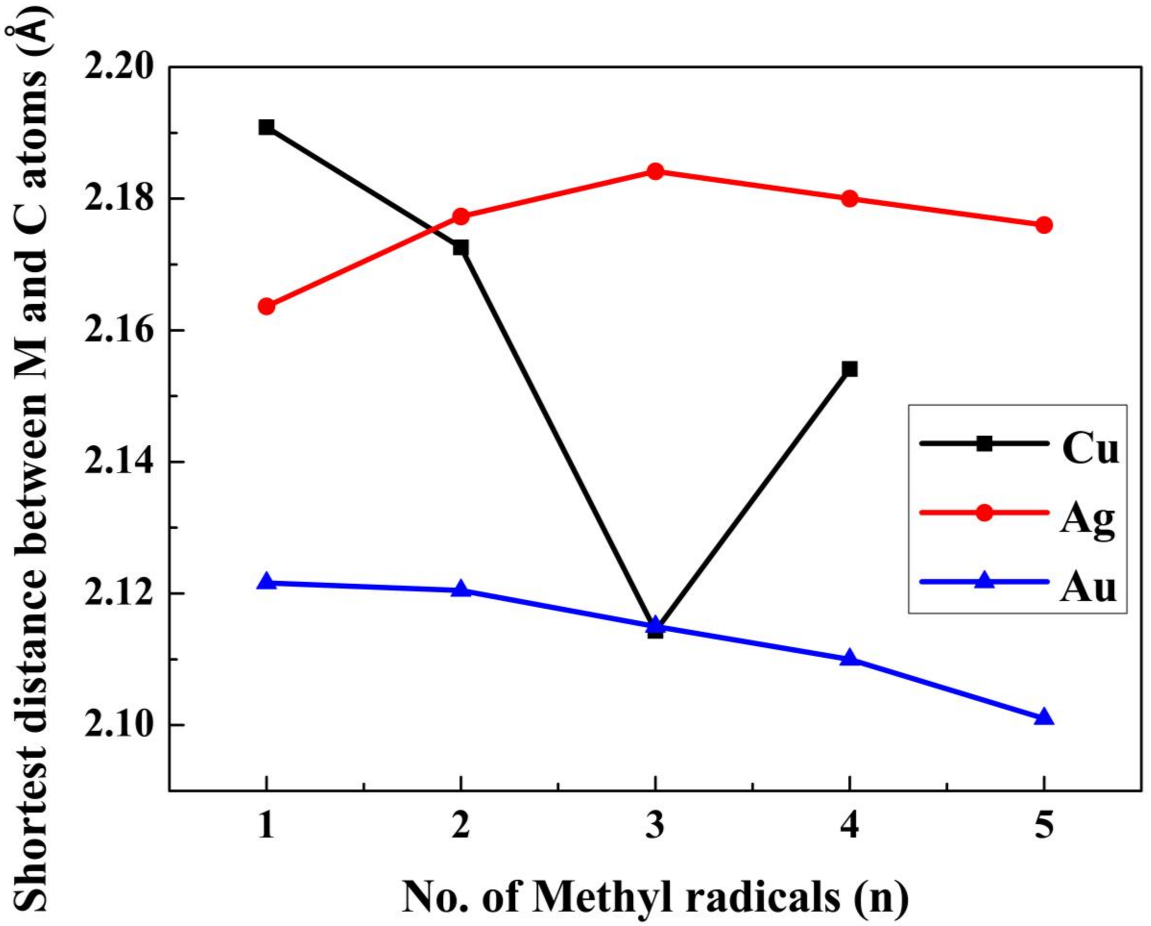

Figure S6: The shortest distance between M and carbon atoms (Å) under different surface coverage in the gaseous phase.

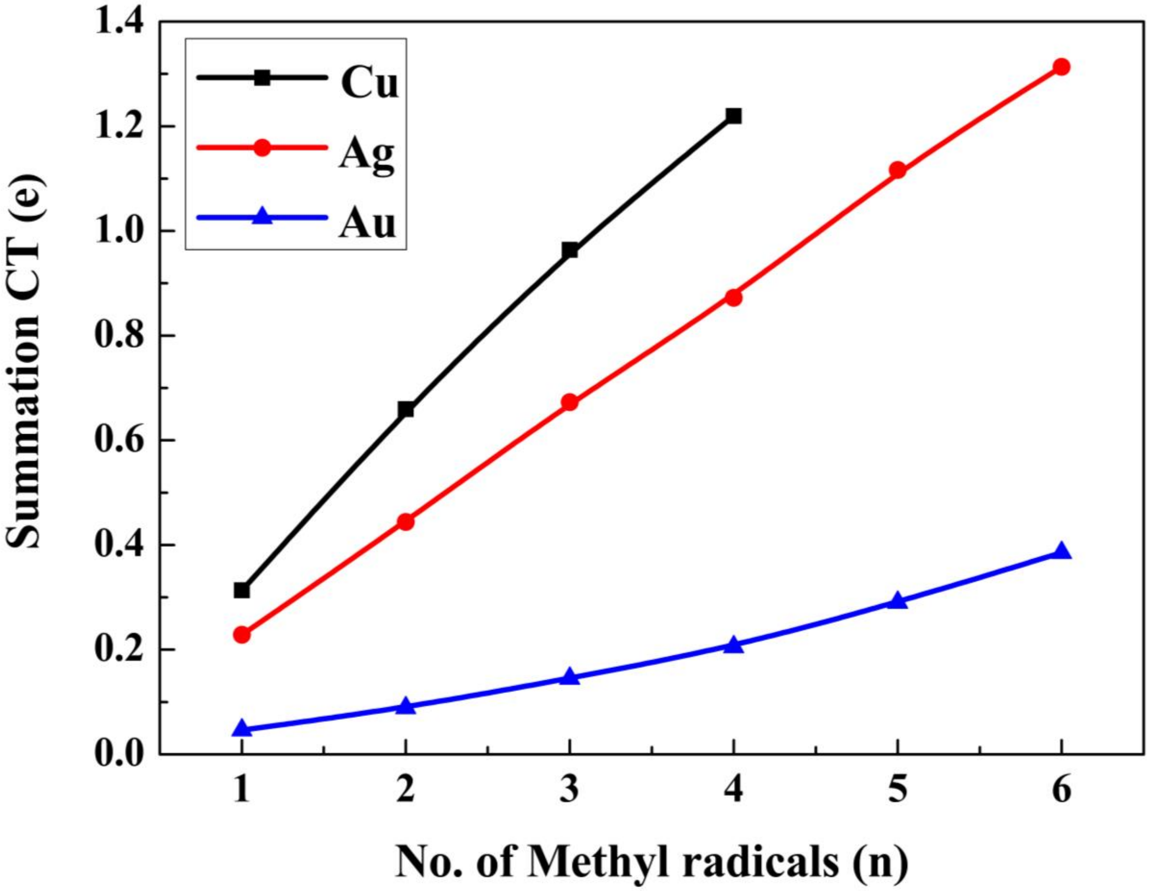

Figure S7: The summation of the charge transfer (CT) of n methyl radicals (n=1-6) on M(111) surfaces in the aqueous phase.

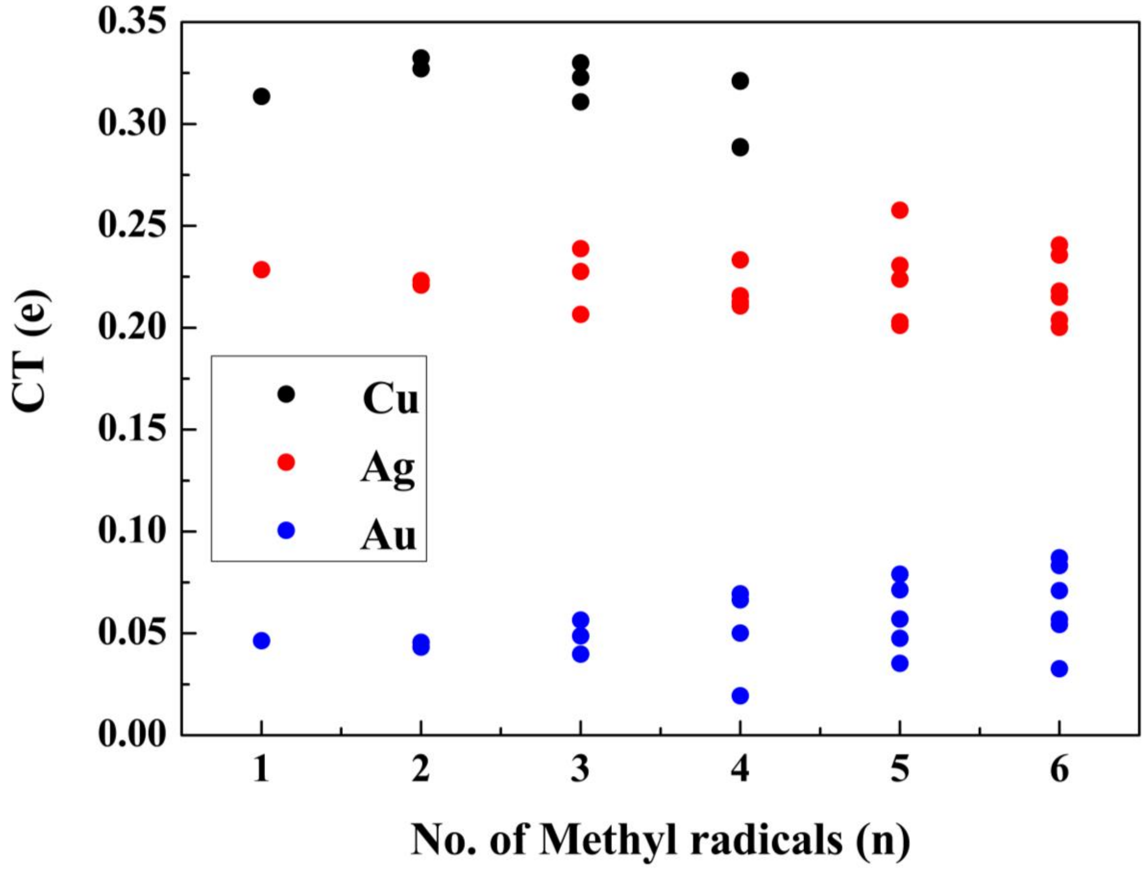

Figure S8: The charge transfer (CT) of every methyl radical on M(111) surfaces in aqueous phase.

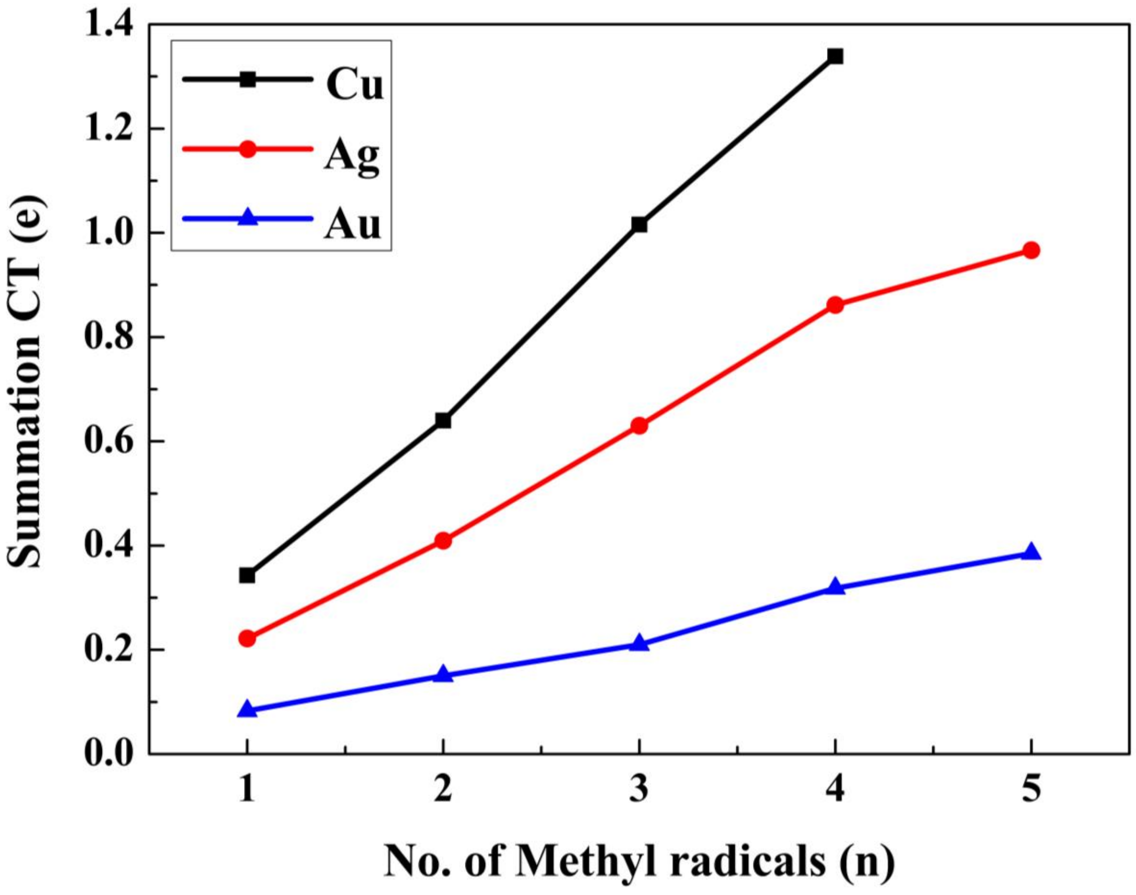

Figure S9: The summation of the charge transfer (CT) of n methyl radicals (n=1-5) on M(111) surfaces in the gaseous phase.

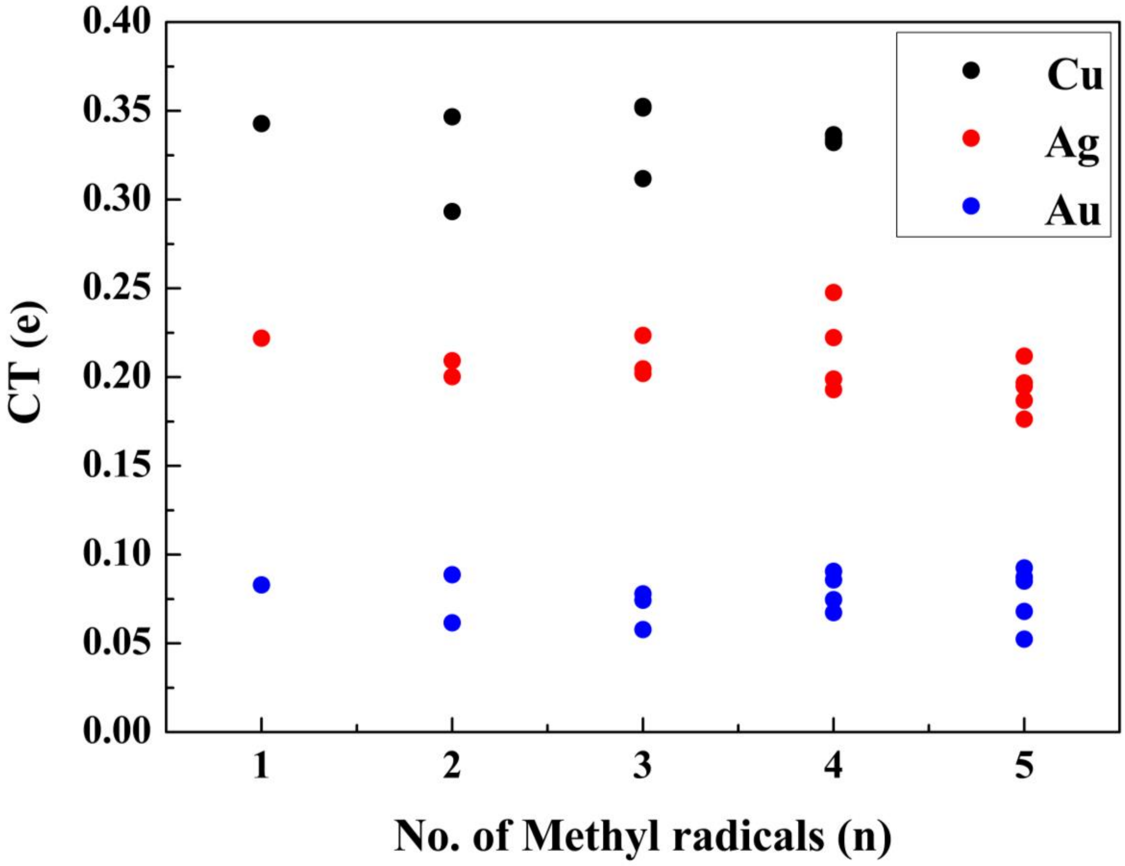

Figure S10: The charge transfer (CT) of every methyl radical on M(111) surfaces in the gaseous phase.

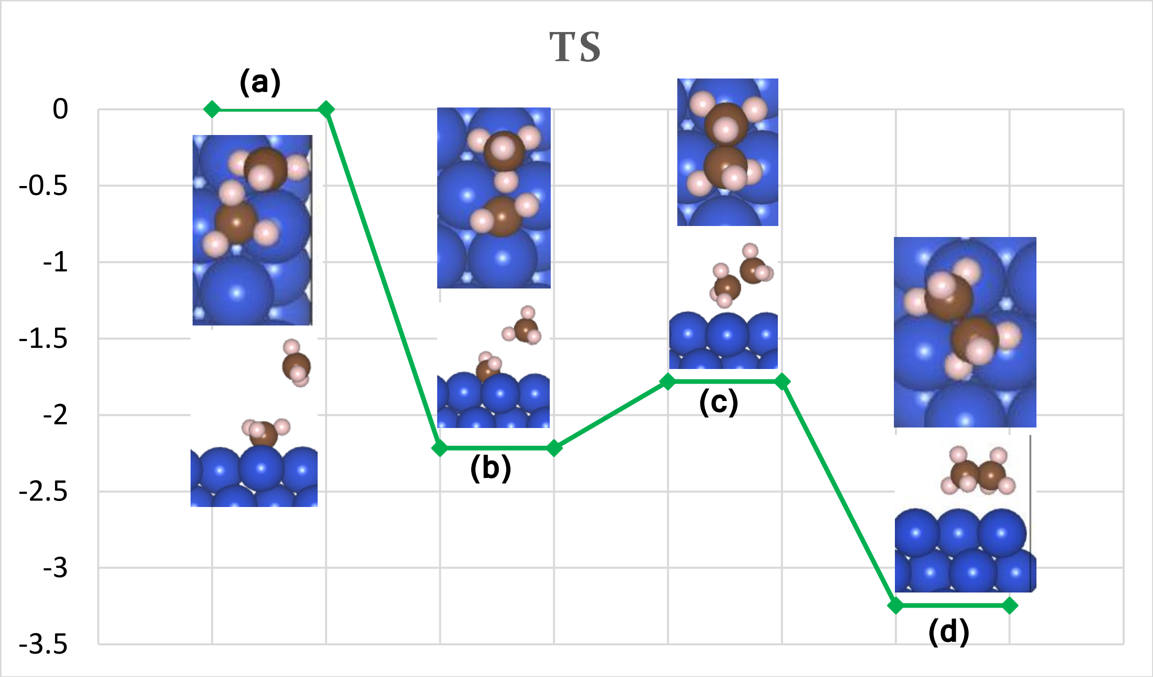

Figure S11: The structural geometry (top and side view) of (a) reactant, (c) transition state, and (d) product ethane production via one adsorbed methyl radical and another one in solution, methane (CH_4) is formed at (b) on the Cu(111) surface for aqueous medium.

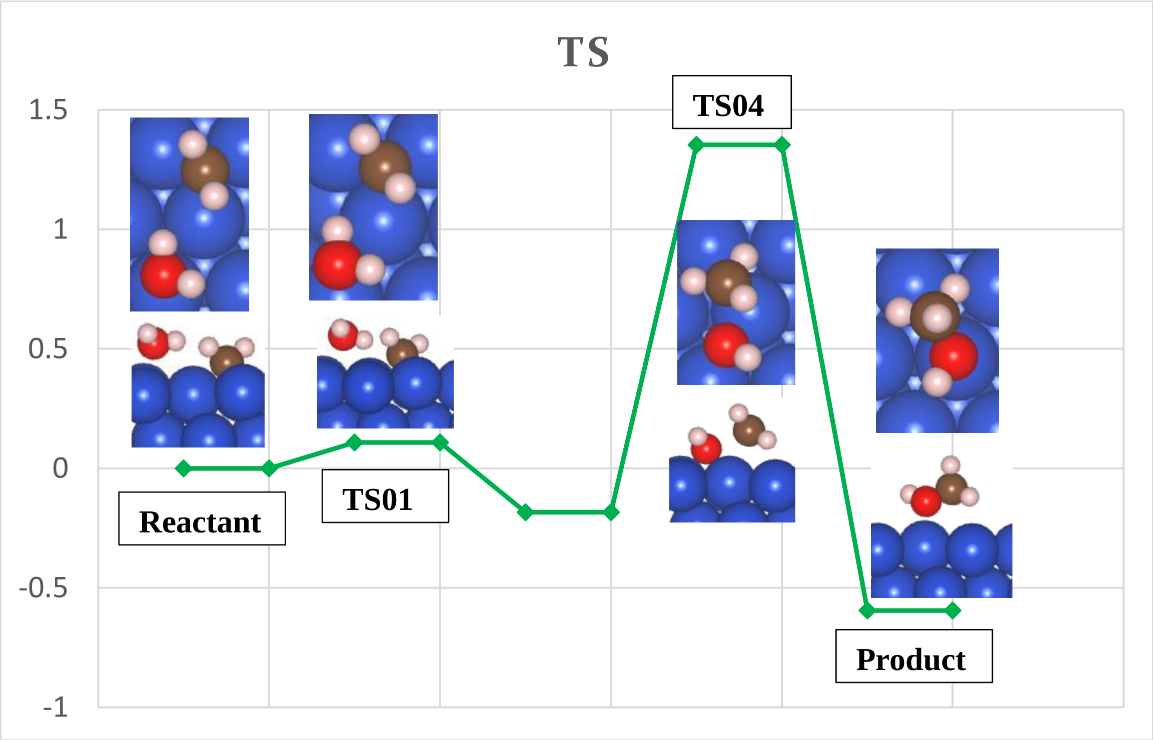

Figure S12: The structural geometry (top and side view) of reactant, transition state, and product for methanol (CH_3OH) production via adsorbed CH_2* and H_2O* on Cu(111) surface.

Tables

Table S1: The adsorption energies (E_{ads}) on the M(111) surfaces at the best adsorption sites for the methyl radical and the ethane in gaseous medium.

**M(111) surfaces**	**E_{ads} (eV)**	
	Best CH_3 adsorption	Best C_2H_6 adsorption
Cu	-2.28	-0.43
Ag	-1.66	-0.16
Au	-2.54	-0.76

Table S2: The charge transfer (e) of a single methyl radical (CH_3) on different adsorption sites on M(111) surfaces.

**M(111) surfaces**	**Aqueous**				**Gaseous**			
	**bridge**	**fcc**	**hcp**	**atop**	**bridge**	**fcc**	**hcp**	**atop**
Cu	0.32	0.31	0.31	0.24	0.35	0.34	0.35	0.27
Ag	0.25	0.26	0.26	0.23	0.26	0.27	0.27	0.22
Au	0.03	0.03	0.05	0.05	0.04	0.05	0.05	0.08

Table S3: The charge transfer (e) of ethane (C_2H_6) on different adsorption sites on M(111) surfaces.

**M(111) surfaces**	**Aqueous**				**Gaseous**			
	**bridge**	**fcc**	**hcp**	**atop**	**bridge**	**fcc**	**hcp**	**atop**
Cu	0.03	0.03	0.03	0.04	0.04	0.04	0.04	0.04
Ag	0.30	0.02	0.03	0.04	0.04	0.04	0.04	0.05
Au	0.01	-0.02	-0.01	0.01	0.01	-0.003	0.01	0.02

Table S4: The optimized geometries (top view) of a single methyl radical adsorbed on Cu(111), Ag(111), and Au(111) surfaces in aqueous phase.

**M(111) surfaces**	**bridge**	**fcc**	**hcp**	**atop**

Cu	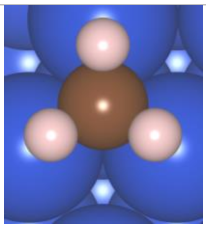	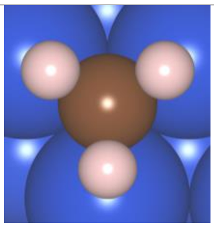	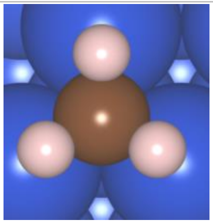	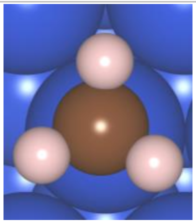
Ag	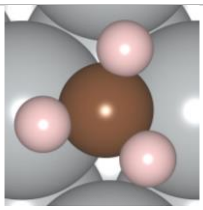	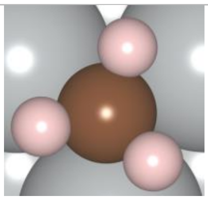	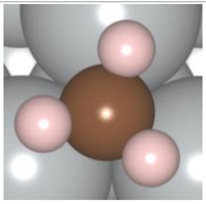	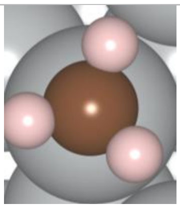
Au	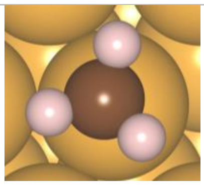	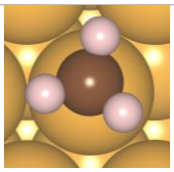	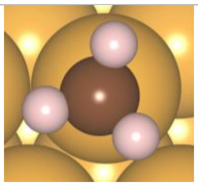	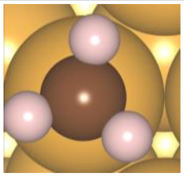

Table S5: The optimized geometries (top view) of a single methyl radical adsorbed on Cu(111), Ag(111), and Au(111) surfaces in the gaseous phase.

**M(111) surfaces**	**bridge**	**fcc**	**hcp**	**atop**
Cu	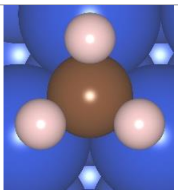	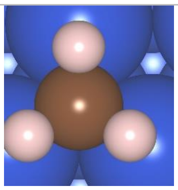	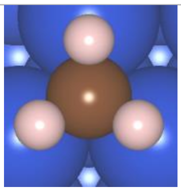	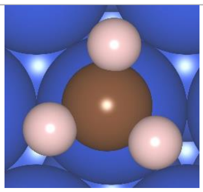
Ag	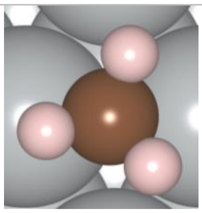	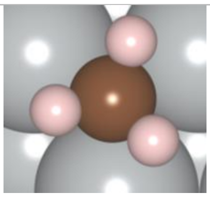	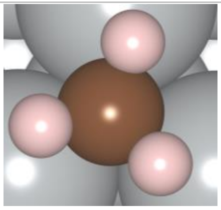	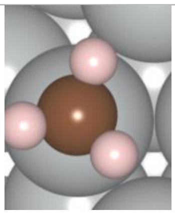

Au	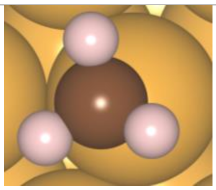	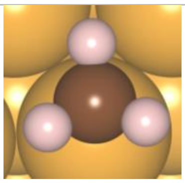	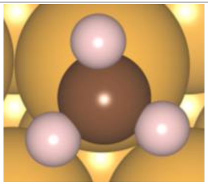	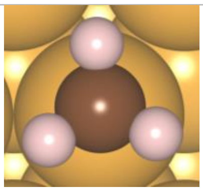

Table S6: The optimized geometries (top view) of ethane adsorbed on Cu(111), Ag(111), and Au(111) surfaces in aqueous phase.

**M(111) surfaces**	**bridge**	**fcc**	**hcp**	**atop**
Cu	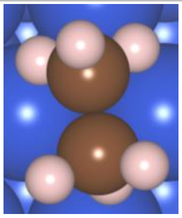	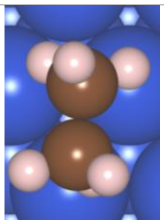	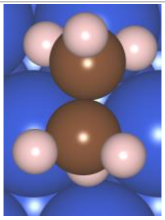	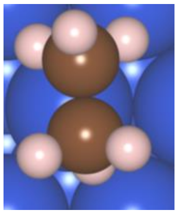
Ag	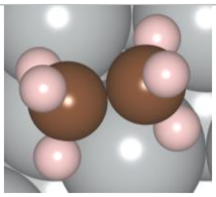	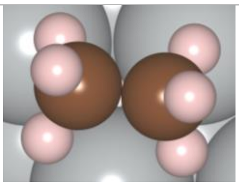	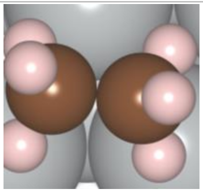	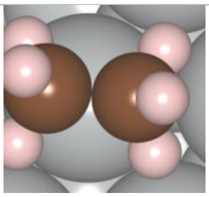
Au	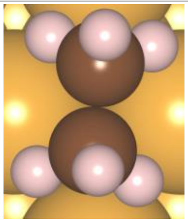	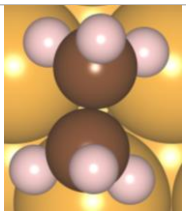	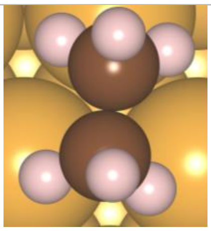	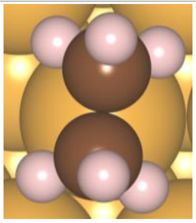

Table S7: The optimized geometries (top view) of ethane adsorbed on Cu(111), Ag(111), and Au(111) surfaces in the gaseous phase.

**M(111) surfaces**	**bridge**	**fcc**	**hcp**	**atop**

Cu	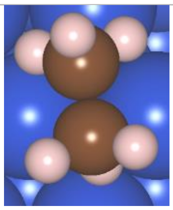	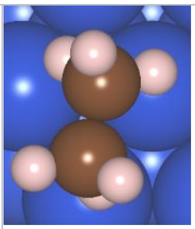	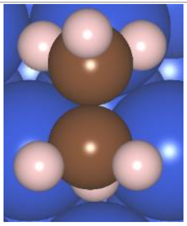	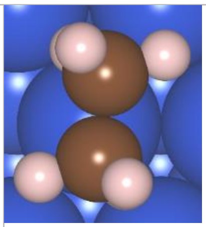
Ag	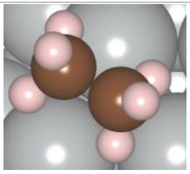	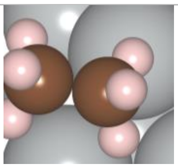	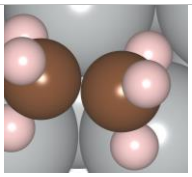	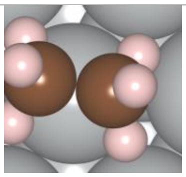
Au	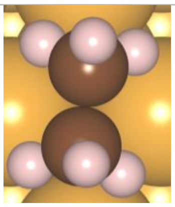	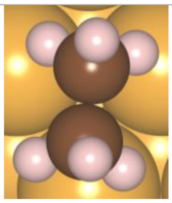	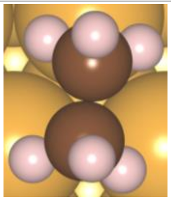	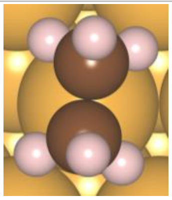

Table S8: Two different scenarios for the adsorption geometries (top view) and adsorption energies of two adsorbed CH_3 radicals on M(111) surfaces in the aqueous phase.

**M(111) surfaces**	**Reactant1**	**Reactant2**
Cu	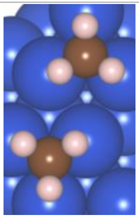	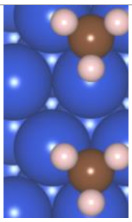
	E_{ads} = -4.60 eV; r(Cu-C) = 2.17 Å; r(C-C) = 3.73 Å	E_{ads} = -4.62 eV; r(Cu-C) = 2.18 Å; r(C-C) = 4.45 Å

Ag	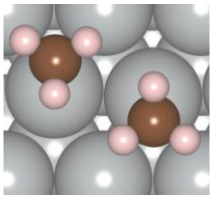	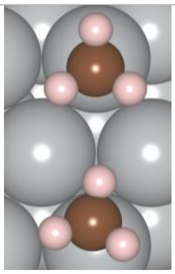
	E_{ads} = -3.86 eV; r(Ag-C) = 2.20 Å; r(C-C) = 3.38 Å	E_{ads} = -3.93 eV; r(Ag-C) = 2.19 Å; r(C-C) = 4.09 Å
Au	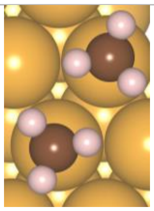	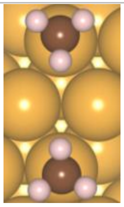
	E_{ads} = -4.98 eV; r(Au-C) = 2.12 Å; r(C-C) = 3.31 Å	E_{ads} = -4.65 eV; r(Au-C) = 2.12 Å; r(C-C) = 5.47 Å

Table S9: Two different scenarios for the adsorption geometries (top view) and adsorption energies of two adsorbed CH_3 radicals on M(111) surfaces in the gaseous phase.

**M(111) surfaces**	**Reactant1**	**Reactant2**
Cu	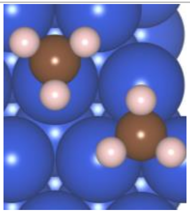	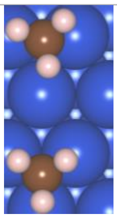
	E_{ads} = -4.52 eV; r(Cu-C) = 2.02 Å; r(C-C) = 3.37 Å	E_{ads} = -4.50 eV; r(Cu-C) = 2.12 Å; r(C-C) = 5.03 Å

Ag	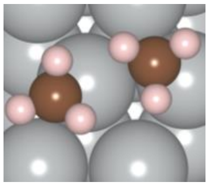	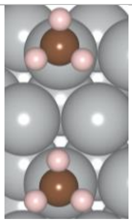
	E_{ads} = -3.33 eV; r(Ag-C) = 2.22 Å; r(C-C) = 3.03 Å	E_{ads} = -3.53 eV; r(Ag-C) = 2.18 Å; r(C-C) = 5.51 Å
Au	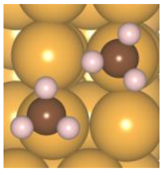	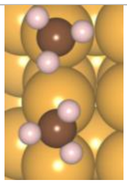
	E_{ads} = -5.54 eV; r(Au-C) = 2.12 Å; r(C-C) = 3.45 Å	E_{ads} = -4.91 eV; r(Au-C) = 2.15 Å; r(C-C) = 3.57 Å

Table S10: The optimized geometries (top view) of n of methyl radical adsorbed on Cu(111), Ag(111) and Au(111) surfaces in aqueous phase.

**No. of methyl radicals (CH_3)**	**Cu(111)**	**Ag(111)**	**Au(111)**
1	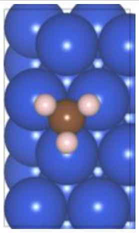	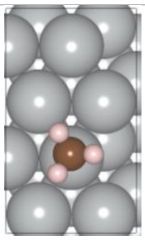	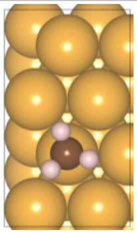
2	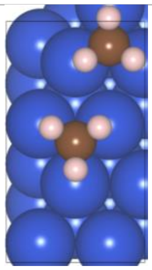	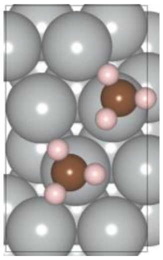	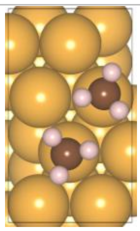

3	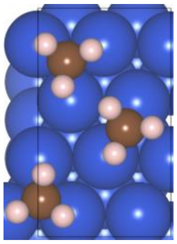	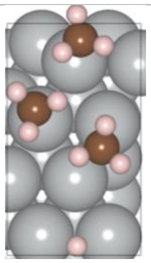	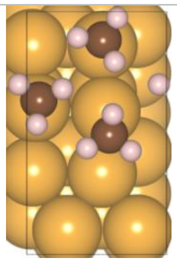
4	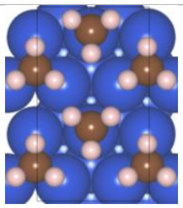	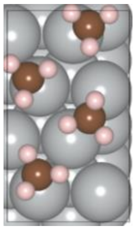	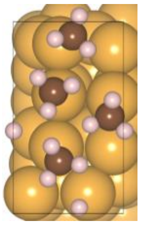
5	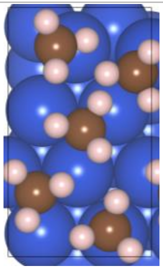	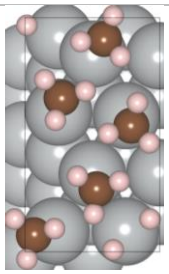	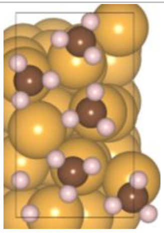
6	–	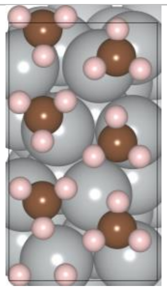	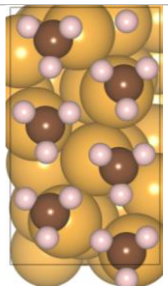
		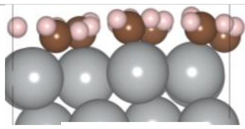 **Side view**	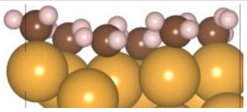 **Side view**

7	–	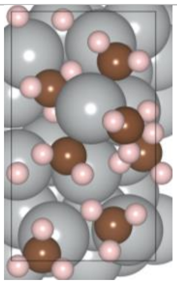	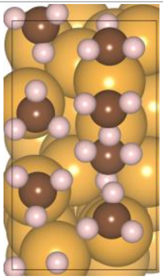
		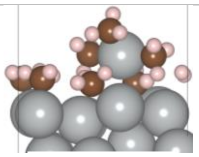 **Side view**	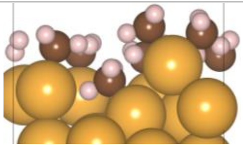 **Side view**

Table S11: The optimized geometries (top view) of a methyl radical adsorbed on Cu(111), Ag(111), and Au(111) surfaces in the gaseous phase.

**No. of methyl radicals (CH_3)**	**Cu(111)**	**Ag(111)**	**Au(111)**
1	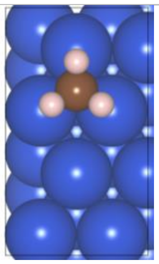	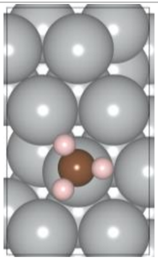	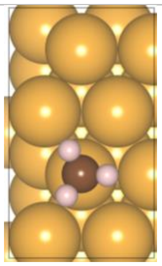
2	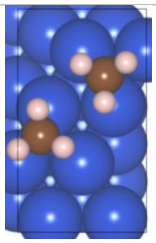	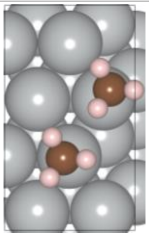	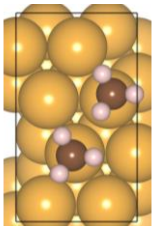
3	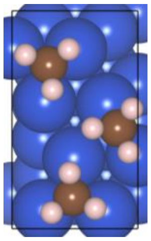	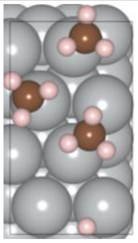	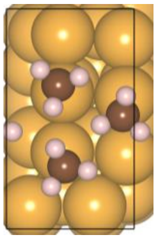

4	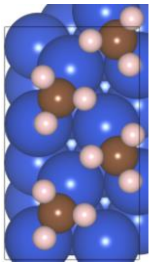	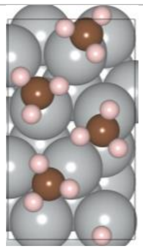	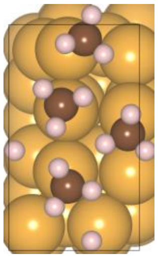
5	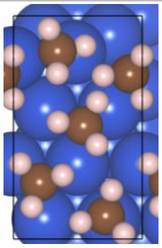	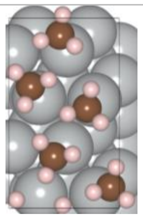	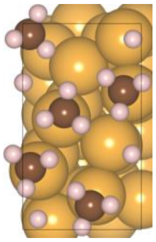
6	–	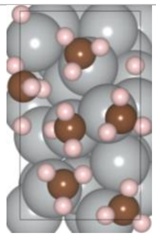	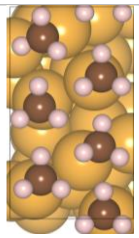
		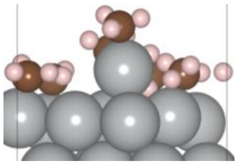 **Side view**	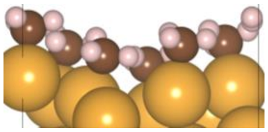 **Side view**
7	–	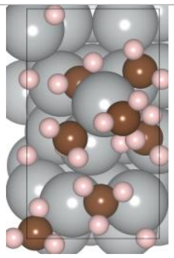	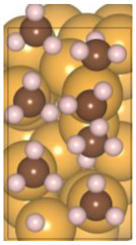
		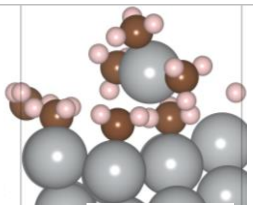 **Side view**	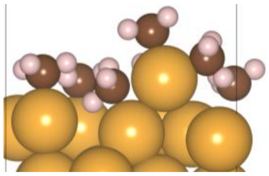 **Side view**

Table S12: The optimized geometries (top view) of initial state (IS), transition state (TS), and product or final state (FS) on Cu(111) surface in the gaseous phase.

**No. of methyl radicals (CH_3)**	**Reactant**	**TS**	**Product**
2	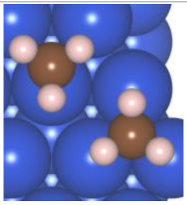	**TS02** 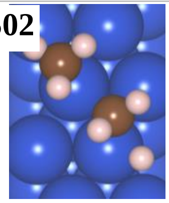	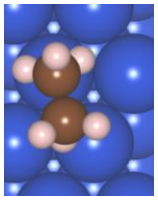
3	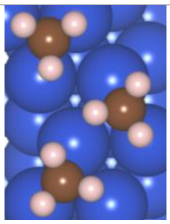	**TS03** 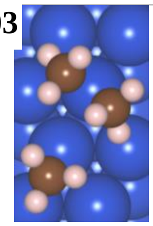	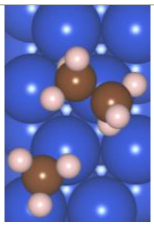
4	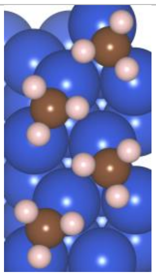	**TS03** 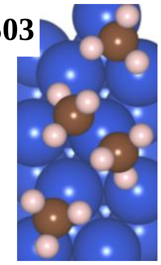	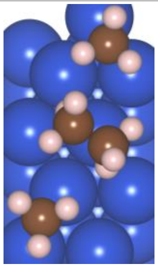

Table S13: The optimized geometries (top view) of reactants, transition state, and product on Ag(111) surface in the gaseous phase.

**No. of methyl radicals (CH_3)**	**Reactant**	**TS**	**Product**

2	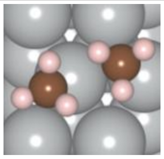	TS02 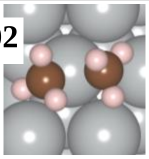	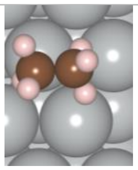
5	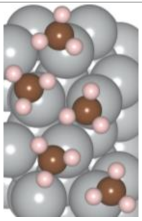	TS03 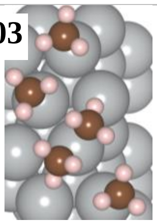	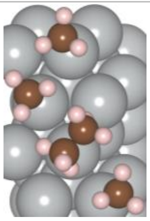

Table S14: The optimized geometries (top view) of reactants, transition state and product on Au(111) surface in the gaseous phase.

No. of methyl radicals (CH_3)	Reactant	TS	Product
2	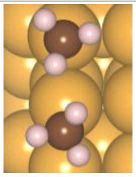	TS03 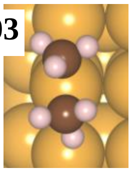	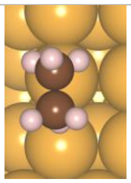
5	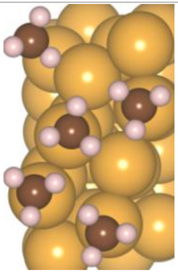	TS02 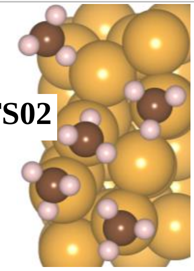	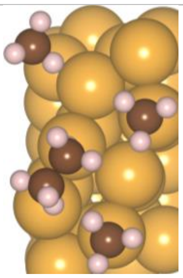

Table S15: The optimized geometries (top and side view) for ethane and methane production via one adsorbed methyl radical and another one in solution to Cu(111) surface in aqueous phase.

Adsorbed on Cu(111) surface	Initial ($CH_3^* + CH_{3(aq)}$)	Intermediate path ($CH_2^* + CH_4^*$)	Intermediate path ($C_2H_6^*$)	Final ($C_2H_6^*$)

Top view	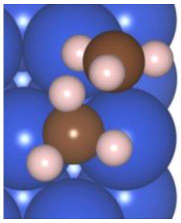	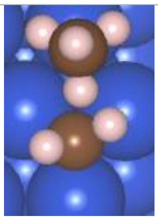	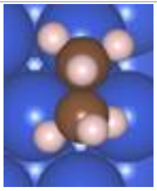	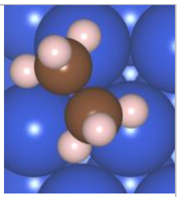
Side view	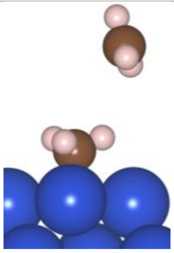	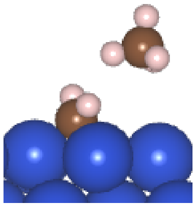	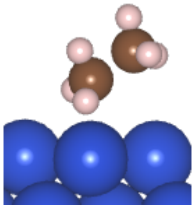	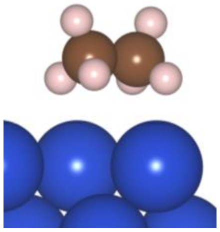

Table S16: The optimized geometries (top view) for the initial state, transition state, and final state for the diffusion of CH_3 on M(111) surfaces in the gaseous phase.

**M(111) surfaces**	**Initial**	**TS**	**Final**
Cu	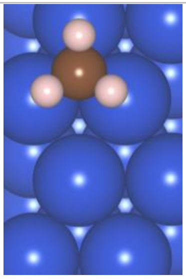	**TS02** 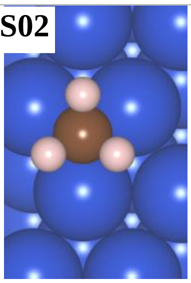	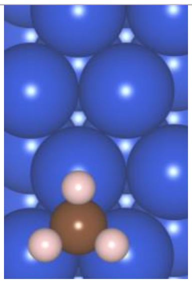
Ag	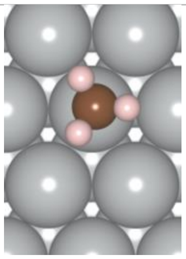	**TS03** 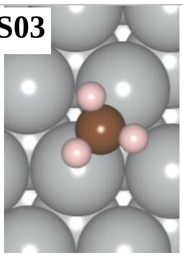	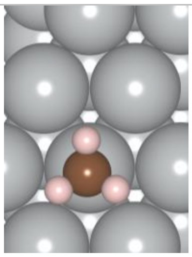

Au	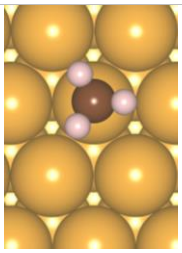	**TS03** 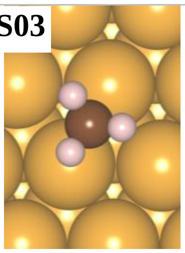	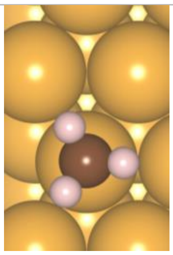

Table S17: The optimized geometries (top and side view) for the diffusion of CH_3 from the solution to Cu(111) surface in the aqueous phase.

Adsorbate on Cu(111) surface	Initial state ($CH_{3(aq)}$)		TS	Final state (CH_3^*)	
	Top view	Side view		Top view	Side view
CH_3	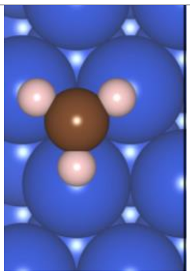	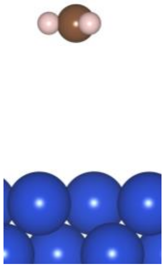	–	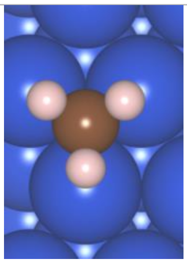	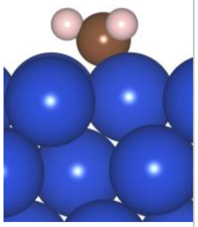

Table S18: The Gibbs free energy ($\Delta G^0_{CH_3(aq)}$) values for adsorption of n (n=1-6) methyl radicals on M(111) surfaces in aqueous solution.

No. of Methyl radicals	$\Delta G^0_{CH_3(aq)}$(eV)		
	Cu(111)	Ag(111)	Au(111)
1	-2.28	-1.87	-2.32
2	-2.27	-1.88	-2.33
3	-2.21	-1.82	-2.27
4	-1.93	-1.84	-2.22
5	0.68	-1.39	-2.02
6	–	-1.10	-1.99

Table S19: The Gibbs free energy ($\Delta G^0_{CH_3(g)}$) values for adsorption of n (n=1-5) methyl radicals on M(111) surfaces in gaseous medium.

**No. of methyl radicals** (CH_3)	$\Delta G^0_{CH_3(g)}$ **(eV)**		
	**Cu(111)**	**Ag(111)**	**Au(111)**
1	-2.28	-1.96	-2.54
2	-2.29	-1.85	-2.34
3	-2.21	-1.81	-2.31
4	-1.84	-1.72	-2.24
5	0.55	-1.31	-2.24

Table S20: The reaction free energy ($\Delta G^0_{C_2H_6(g)}$) values for ethane production, for methyl radicals on M(111) surfaces for the ethane (C_2H_6) evolution in the gaseous medium.

**No. of methyl radicals** (CH_3)	$\Delta G^0_{C_2H_6(g)}$ **(eV)**		
	**Cu(111)**	**Ag(111)**	**Au(111)**
2	-0.38	-1.20	-0.10
3	-0.44	-1.35	-0.34
4	-0.89	-1.48	-0.43
5	—	-1.98	-0.51

Table S21: The activation barrier energy (E_a) values of n methyl radicals on M(111) surfaces for the ethane (C_2H_6) evolution in gaseous medium.

**No. of methyl radicals** (CH_3)	E_a **(eV)**		
	**Cu(111)**	**Ag(111)**	**Au(111)**
2	1.47	0.75	2.43
4	1.40	—	—
5	—	0.72	2.09
6	—	—	—

Table S22: The activation energy barrier (E_a) values for the diffusion of methyl radicals on M(111) surfaces in the gaseous medium.

**Metals**	**Diffusion barrier (E_a) (eV)**

Cu(111)	0.09
Ag(111)	0.07
Au(111)	0.55

VASP INCAR File

```
Job Title
#Start parameters
INIWAV = 1
ISTART = 0
ICHARG = 2

#Electronic Relaxation
ALGO = FAST
AMIX = 0.1
BMIX = 0.001
ENMAX = 500
EDIFF = 1E-05
ISMEAR = 1
NELM = 120
IVDW = 1          #dispersion correction
LSOL = .TRUE.     #solvation

#Ionic Relaxation
IBRION = 2
```

```
NSW = 500
ISIF = 2

#Performance optimization
NCORE = 8
LREAL = Auto
#Phonon Calculation (changes and additions in the INCAR)
NFREE = 2
POTIM = 0.15   #Diffrent values are used (Section 2)
IBRION = 6
NSW = 1

#DOS calculation (changes and additions in the INCAR)
ISMEAR = -5
IBRION = -1
ICHARG = 11
LORBIT = 11
NEDOS = 500
NSW = 0
#TS calculation (changes and additions in the INCAR)
IBRION = 3
POTIM = 0
IOPT = 3
LCLIMB = .TRUE.
IMAGES = 5
TIMESTEP = 0.01
ENMAX = 400
```

```
SIGMA = 0.05

SPRING = -5

LSCALAPACK = .FALSE.
```
